# Potent telomerase activators from a novel sapogenin via biotransformation utilizing *Camarosporium laburnicola*, an endophytic fungus

**DOI:** 10.1186/s12934-023-02069-3

**Published:** 2023-04-06

**Authors:** Melis Küçüksolak, Sinem Yılmaz, Petek Ballar-Kırmızıbayrak, Erdal Bedir

**Affiliations:** 1 Department of Bioengineering, Izmir Institute of Technology, Urla, 35430 İzmir, Türkiye Turkey; 2 Department of Biotechnology, Graduate School of Natural and Applied Sciences, Ege University, Bornova, İzmir, Türkiye Turkey; 3 Department of Bioengineering, Faculty of Engineering, University of Alanya Aladdin Keykubat, Antalya, Türkiye Turkey; 4 Department of Biochemistry, Faculty of Pharmacy, Ege University, Bornova, 35100 İzmir, Türkiye Turkey

**Keywords:** Biotransformation, Endophytic Fungi, Sapogenin, Telomerase activator, Anti-aging

## Abstract

**Background:**

Cycloartane-type triterpenoids possess important biological activities, including immunostimulant, wound healing, and telomerase activation. Biotransformation is one of the derivatization strategies of natural products to improve their bioactivities. Endophytic fungi have attracted attention in biotransformation studies because of their ability to perform modifications in complex structures with a high degree of stereospecificity.

**Results:**

This study focuses on biotransformation studies on cyclocephagenol (**1**), a novel cycloartane-type sapogenin from *Astragalus* species, and its 12-hydroxy derivatives (**2** and **3**) to obtain new telomerase activators. Since the hTERT protein levels of cyclocephagenol (**1**) and its 12-hydroxy derivatives (**2** and **3**) on HEKn cells were found to be notable, biotransformation studies were carried out on cyclocephagenol and its 12-hydroxy derivatives using *Camarosporium laburnicola*, an endophytic fungus isolated from *Astragalus angustifolius*. Later, immunoblotting and PCR-based ELISA assay were used to screen starting compounds and biotransformation products for their effects on hTERT protein levels and telomerase activation. All compounds showed improved telomerase activation compared to the control group.

**Conclusions:**

As a result of biotransformation studies, seven new metabolites were obtained and characterized, verifying the potential of *C. laburnicola* as a biocatalyst. Additionally, the bioactivity results showed that this endophytic biocatalyst is unique in transforming the metabolites of its host to afford potent telomerase activators.

**Supplementary Information:**

The online version contains supplementary material available at 10.1186/s12934-023-02069-3.

## Background

Modification of natural products occurs either by chemical reactions or by biotransformation reactions. Biotransformation is the biochemical reactions of living systems or their components (enzymes) to alter molecules. It is an effective tool in obtaining molecules that are difficult to prepare by conventional synthetic methods and has a wide range of uses. In the pharmaceutical industry, microbial biotransformation has been utilized in the enzymatic transformation to synthesize chiral intermediates and end products [1–4].

There are many applications of biotransformation in drug discovery and development studies, including the synthesis of drug metabolites for estimation of mammalian metabolism, lead optimization, and the development of metabolite libraries from highly diverse lead compounds for structure-activity relationship (SAR) and bioactivity screening studies [5]. In addition to overcoming the difficulties encountered in chemical synthesis, microbial biotransformation is effectively used to increase/reduce the bioactivity/toxicity profiles of drug precursor molecules and to generate molecular libraries for structure-activity studies with their highly diverse enzyme systems [3, 6–9].

Cycloartane-type triterpenoids are produced only by photosynthetic eukaryotes. In the plant kingdom, these metabolites are not as common as other triterpenoids (oleanane, ursane, steroidal triterpenoids). The plants richest in cycloartane-type of compounds are *Astragalus* species [10]. Nearly 650 cycloartane-type metabolites were identified, and almost half were obtained from *Astragalus* species [11, 12]. The biological activities of cycloartanes and their semi-synthetic derivatives indicate their wide range of bioactivities, including immunostimulant [13–15], anti-protozoal [16], antiviral [17], cytotoxic [18, 19], cardiotonic [20], wound healing [21] and adjuvant [22–24] activities. The most important development in the last ten years for cycloartanes was the discovery of cycloastragenol as a potent telomerase activator [25]. Since 2007, cycloastragenol, present only in *Astragalus* species, has been in the market as an anti-aging dietary supplement with the trade name TA-65.

Telomeres are specialized structures consisting of TTAGGG tandem repeat sequences at the ends of chromosomes. They protect chromosome ends from fusion and degradation and shorten over time in dividing somatic cells [26–28]. Telomerase is a cellular “reverse transcriptase” (TERT, telomerase reverse transcriptase) enzyme that uses the relevant RNA component (Terc, telomerase RNA component) as a template and helps repair telomere ends by adding TTAGGG repeats [29].

Telomerase activation is a potential target for treating diseases associated with telomere loss, as telomere shortening results from the biological aging process and is also considered a risk factor for many age-related diseases. In addition, conditions associated with telomere shortening are not only age-related. Hence, telomerase activation *in* *vivo* has significant potential for treating many chronic and degenerative diseases [30–35].

Cycloastragenol and its derivatives have been at the center of nature-based regenerative medicine research as well its utilization in degenerative diseases is a hot topic [30–34, 36]. Also, the entry of cycloastragenol and its derivatives into clinical trials for Alzheimer’s disease (NCT number: 02531334) and metabolic syndrome (NCT number: 02530255) is an indication of the potential of telomerase activators toward degenerative diseases. In 2022, two new clinical studies were initiated using *Astragalus membranaceus* in cognitive impairment and parkinsonism (NCT number: 05578443 and 05506891, respectively).

In our previous studies, biotransformation studies were carried out on cycloartanes using endophytic fungi isolated from the tissues of *Astragalus* species, and the effects of the metabolites on telomerase activation were investigated. Cycloastragenol, cyclocanthogenol, and semi-synthetic derivatives astragenol and 20(27)-octanor cycloastragenol, the main triterpenoids in *Astragalus*, were used as starting molecules. In the telomerase activation screening panel using obtained metabolites, 16 compounds showed activity ranging from 1.2 to 11.3-fold at doses of 0.5 to 300 nM compared to the control cells treated with DMSO [37–39]. The significant modifications leading to the increase in activity were the oxidation of C-3(OH), conversion of ring A to 7-membered lactone, and the formation of 3(4)-seco structures via cleavage of the ring A, catalyzed by *Camarosporium laburnicola*. Another substantial modification was the hydroxylation of C-12 by the P450 monooxygenase system of *Alternaria eureka*.

Cyclocephagenol (**1**) is another major sapogenin in Turkish *Astragalus* species with a tetrahydropyran side chain in the cycloartane skeleton. In a recent study, we modified this sapogenin using *Alternaria eureka* to obtain new neuroprotective agents. As a result, potent metabolites exhibited bioactivities at the nanomolar range [40]. However, bioactivities of **1** or its conceivably effective metabolites (**2** and **3**: 12-hydroxy derivatives) on telomerase activation have never been screened. To see the potential of these compounds (**1**–**3**) towards telomerase activation, first, hTERT (human Telomerase Reverse Transcriptase) protein expression levels were evaluated. All compounds increased hTERT protein expression; therefore, we decided to perform a modification study on these compounds utilizing *C. laburnicola*, a well-proven biocatalyst to afford bioactive metabolites [37–39]. Later, we screened biotransformation products for their effects on telomerase activation by immunoblotting and PCR-based ELISA assay.

## Results

### Effect of cyclocephagenol (1), 12α-hydroxycyclocephagenol (2), and 12β-hydroxycyclocephagenol (3) on hTERT expression

**Fig. 1:**
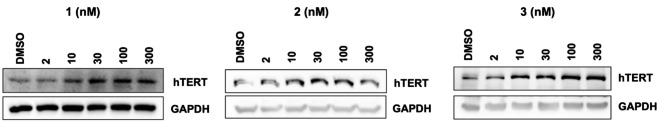
Effects of **1**, **2**, and **3** on hTERT protein level

As hTERT protein level is a preliminary indicator of probable telomerase activation, compounds **1**, **2**, and **3** were screened for their effects on hTERT expression by Western Blot. All three metabolites were found to increase the hTERT protein levels compared to the DMSO-treated control group. While **1** and **3** increased the hTERT protein levels in a dose dependent-manner, metabolite **2** enhanced hTERT protein levels at 10, 30, and 100 nM concentrations (Fig. 1).

Based on the promising activities of these metabolites, biotransformation studies on **1**, **2**, and **3** were carried out to enrich our molecule library to obtain potent telomerase activators and investigate structure-activity relationships.

### Biotransformation of cyclocephagenol (1) and 12α-hydroxycyclocephagenol (2) by *Camarosporium laburnicola*

**Fig. 2:**
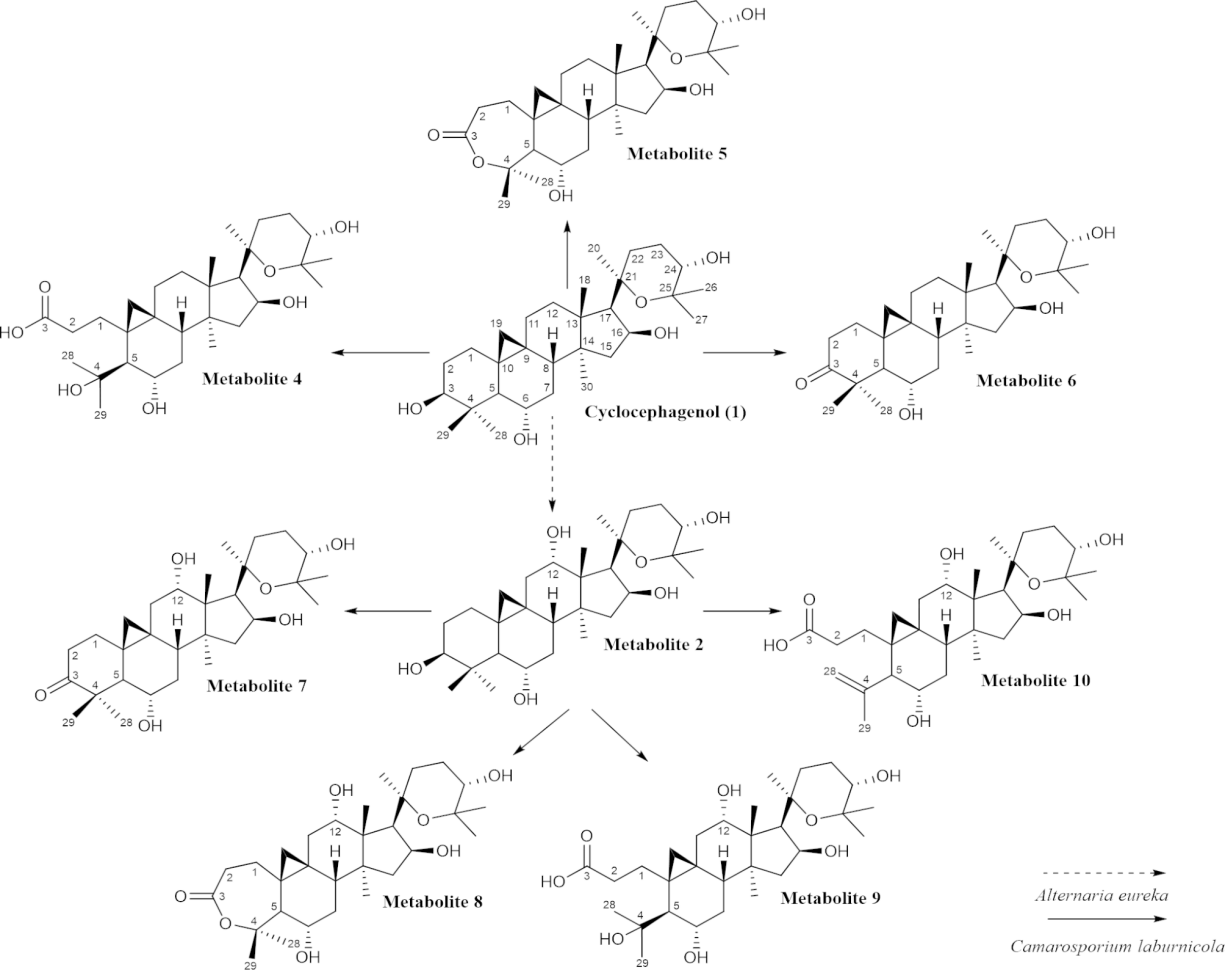
Biotransformation products of **1** and **2** by *Camarosporium laburnicola*

The biotransformation of cyclocephagenol with the endophytic fungus *C. laburnicola* for four days afforded three metabolites (**4–6**), while the biotransformation studies performed on 12α-hydroxycyclocephagenol by *C. laburnicola* for 30 days yielded four metabolites (**7–10**) (Fig. 2). In the case of 12β-hydroxycyclocephagenol (**3**), metabolite production was relatively low. No further attempt was made to isolate its metabolites.

The structures of the compounds were established by 1D-, 2D-NMR, and HR-MS analyses. Comparison of the NMR data for **4**–**6** with those of **1** and **7**–**10** with those of **2** revealed the presence of the same partial structures for the B → E rings [40]. These observations suggested that A ring biotransformation occurred in metabolites **4**–**10**. For metabolites **7**–**10**, α-hydroxylation at C-12 was confirmed using 1D- and 2D-NMR spectra of these compounds.

HR-ESI-MS spectrum of **4** showing a major ion peak at *m/z* 545.34691 ([M + Na]^+^) indicated a molecular formula of C_30_H_50_O_7_. In the ^13^C-NMR spectrum, the oxymethine signal belonging to C-3 was lost, and a carboxyl signal was deduced from δ_C_ 176.0 resonance. In addition, a quaternary carbon adjacent to oxygen (δ_C_ 75.7) was noticed. When five out of six degrees of unsaturation accounted for the ring systems (rings B → E and the cyclopropane) was assessed with the presence of a carboxyl group, revealing an additional unsaturation, a cleavage for ring A was evident. In the HMBC spectrum, the long-distance correlations from H-5, H_3_-28, and H_3_-29 to δ_C_ 75.7 justified the hydroxylation at C-4. Additionally, a downfield shift of H_2_-2 signals (δ_H_ 2.96, m, H-2a; δ_C_ 2.60, m, H-2b) and the long-distance correlations from H-2 and H-1 to the carboxyl signal proved the structure being 3(4)-seco-cyclocephagenol. Based on these results, the structure of **4** was established as 20,25-epoxy-6α,16β,24α-trihydroxy-3,4-secocycloartan-3-oic acid.

The molecular formula of **5** was established as C_30_H_48_O_6_ by HR-ESI-MS analysis (*m/z* 527.33635 [M + Na]^+^). From detailed inspection of 1D- and 2D-NMR spectra, long-range correlations from two methylene groups (H_2_-2 and H_2_-1) to the carbonyl signal at 174.3 ppm suggested a ring opening for **5** at first glance, as in **4**. However, an oxygenated carbon was observed at 85.6 ppm in the ^13^C-NMR spectrum. In the HMBC spectrum, long-range correlations from two methyl groups (δ_H_ 1.71 and 1.49) to δ_C_ 85.6 implied that it was C-4. The downfield shift of C-4 resonance (9.9 ppm) compared to metabolite **4** (δ_C_ 75.7) and previously reported metabolites of our group ascertained that C-4 was in a 7-membered lactone ring system [38, 41]. As a result, **5** was elucidated as 20,25-epoxy-6α,16β,24α-trihydroxycycloartan-3-olide.

The metabolite **6** gave a molecular formula of C_30_H_48_O_5_ based on the HR-ESI-MS data (*m/z* 511.33940 [M + Na]^+^). Apart from the oxymethine signals of C-3, the characteristic signals of the starting molecule **1** were present for **6**. In the ^13^C-NMR spectrum, the resonance at 216.6 ppm was obvious, implying a biotransformation of C-3 secondary alcohol to a ketone via an oxidation reaction. Accordingly, this assumption was substantiated by long-range correlations between H-5/H_3_-28/H_3_-29 and C-3 (δ_C_ 216.6) in the HMBC spectrum. As a result, the structure of **6** was determined to be 20,25-epoxy-6α,16β,24α-trihydroxycycloartan-3-one.

The metabolite **7**, the first metabolite of **2** (12α-hydroxycyclocephagenol), gave a molecular formula of C_30_H_48_O_6_ based on the HR-ESI-MS data (*m/z* 503.33835 [M - H]^−^). Apart from the proton and carbon resonances deriving from ring A, the characteristic signals of **2** were present for **7**. In the ^13^C-NMR spectrum, the resonance at 217.4 ppm was obvious, implying a biotransformation of C-3 secondary alcohol to a ketone as in **6**. The long-range HMBC correlations from H-2b, H_3_-28, and H_3_-29 (δ_H_ 2.43, 1.78, and 1.50, respectively) to C-3 (δ_C_ 217.4) verified the oxidation location. As a result, the structure of **7** was determined to be 20,25-epoxy-6α,12α,16β,24α-tetrahydroxycycloartan-3-one.

The HR-ESI-MS spectrum of **8** showed a major ion peak at *m/z* 519.33289 [M - H]^−^ (C_30_H_48_O_7_). Initial inspection of the ^1^H-NMR spectrum of **8** revealed the absence of characteristic H-3 resonance. The HMBC spectrum showed a cross-peak between the H-1a signal (δ_H_ 1.42) and a carbonyl carbon at 173.6 ppm. From inspection of the ^13^C-NMR and HMBC spectra, the long-range correlations of two methyl groups (δ_H_ 1.94 and 1.70) and H-5 (δ_H_ 2.44) with an oxygenated carbon at δ_C_ 85.3 was observed. Thus, the resonance of δ_C_ 85.3 was readily assigned to C-4. Comparing the chemical shifts and coupling constants with those of **5**, it was ascertained that **8** also had a 7-membered lactone ring system in ring A. As a result, **8** was elucidated as 20,25-epoxy-6α,12α,16β,24α-tetrahydroxycycloartan-3-olide.

HR-ESI-MS spectrum of **9** showing a major ion peak at *m/z* 537.34344 ([M - H]^−^) indicated a molecular formula of C_30_H_50_O_8_. In the ^13^C-NMR spectrum, the oxymethine signal belonging to C-3 was lost, and a carboxyl signal was deduced from δ_C_ 179.5 resonance. In the HMBC spectrum, H_2_-1 displayed cross-peaks with the carboxyl signal at δ_C_ 179.5, confirming the presence of the carboxyl group at C-3. In addition, a low-field carbon adjacent to oxygen (δ_C_ 76.4) was noticed. In the HMBC spectrum, the long-distance correlations from H-5, H_3_-28 and H_3_-29 to δ_C_ 76.4 helped us to assign it as C-4. These data implied a ring cleavage in ring A, as in **4.** The resemblance of the spectral data of **9**, deduced from 1D- and 2D-NMR spectra, with those of **4** and **2** was evident for the characteristic 3(4)-seco structure with a carboxyl group at C-3 and hydroxyl functionality at C-4 establishing the structure of **4**. Based on these results, the structure of **9** was established as 20,25-epoxy-6α,12α,16β,24α-tetrahydroxy-3,4-secocycloartan-3-oic acid.

In the HR-ESI-MS spectrum of **10**, the major ion peak at *m/z* 519.33122 [M - H]^−^ confirmed the molecular formula as C_30_H_48_O_7_. The ^1^H and ^13^C-NMR spectra of **10** implied a ring cleavage as in compound **9**. The resonances observed at δ_H_ 5.21 and 5.09 suggested an exocyclic methylene group corresponding to carbon at δ_C_ 115.3 in the HSQC spectrum. In addition, one of the two methyl groups at C-4 disappeared, and the other shifted to a low field (δ_H−29_ 1.89). The HMBC spectrum displayed cross-peaks from H_3_-29 (δ_H_ 1.89) to C-28 (δ_C_ 115.3), C-4 (δ_C_ 147.1), and C-5 (δ_C_ 55.7). This indicated that an A-ring cleavage was followed by a dehydration reaction yielding a C-4(28) disubstituted double bond system. Accordingly, the ^1^H-^1^H COSY spectrum revealed a spin system of H-28a (δ_H_ 5.21) → H-28b (δ_H_ 5.09) → H_3_-29 (δ_H_ 1.89) and justifying this assignment. Unexpectedly, the cross-peaks from H_2_-1 and H_2_-2 to the carboxyl carbon at δ_C_ 177.3 was not noticeable in the HMBC spectrum. Nevertheless, resemblance of the spectral data of **10**, deduced from 1D- and 2D-NMR spectra, with those of previously reported, compound was evident for the characteristic 3,4-seco structure with an isopropenyl group extending from C-5. Consequently, a new 3(4)-seco structure of 12α-hydroxycyclocephagenol with an isopropenyl group extending from C-5 was established. Based on these results, the structure of **10** was established as 20,25-epoxy-6α,12α,16β,24α-tetrahydroxy-3,4-secocycloartan-4(28)-en-3-oic acid.

### The effects of biotransformation products on hTERT protein levels

**Fig. 3:**
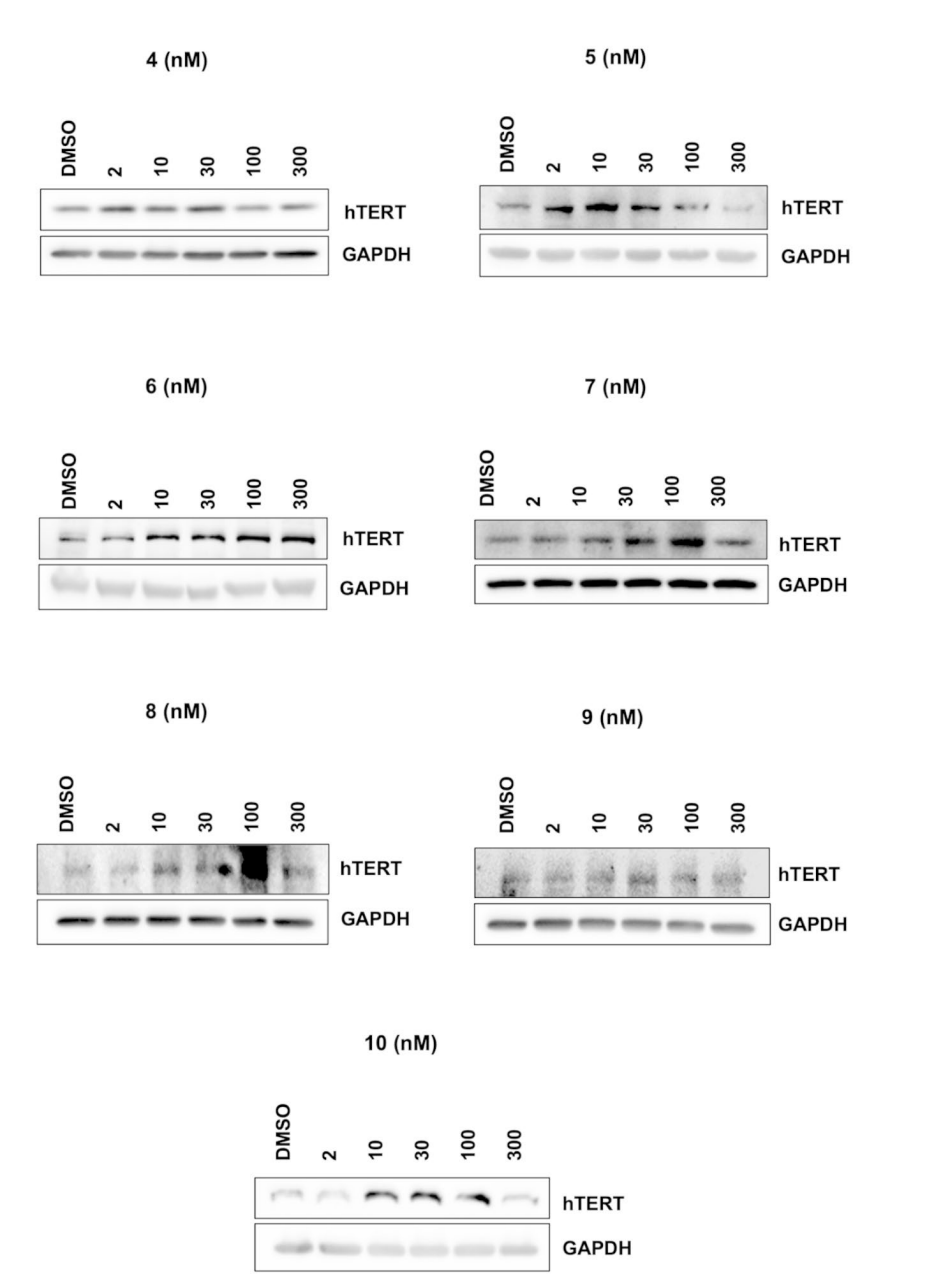
Effects of biotransformation products (**4**–**10**) on hTERT protein level

In preliminary screening, Western blot experiments were performed to examine the effect of biotransformation products on the hTERT protein level, which is a reverse transcriptase subunit of the telomerase enzyme. While metabolites **4** and **5** enhanced hTERT protein levels at lower concentrations as 2, 10, and 30 nM, metabolites **7**–**10** increased protein levels at 10, 30, and 100 nM concentrations—besides, metabolite **6** increased hTERT protein levels in a dose-dependent manner (Fig. 3).

### Determination of telomerase enzyme activity by using TeloTAGGG Assay

After evaluating hTERT protein levels via Western Blot studies, the concentrations that increased protein levels were selected and investigated in terms of telomerase enzyme activity via Telomerase PCR ELISA assay.

**Fig. 4:**
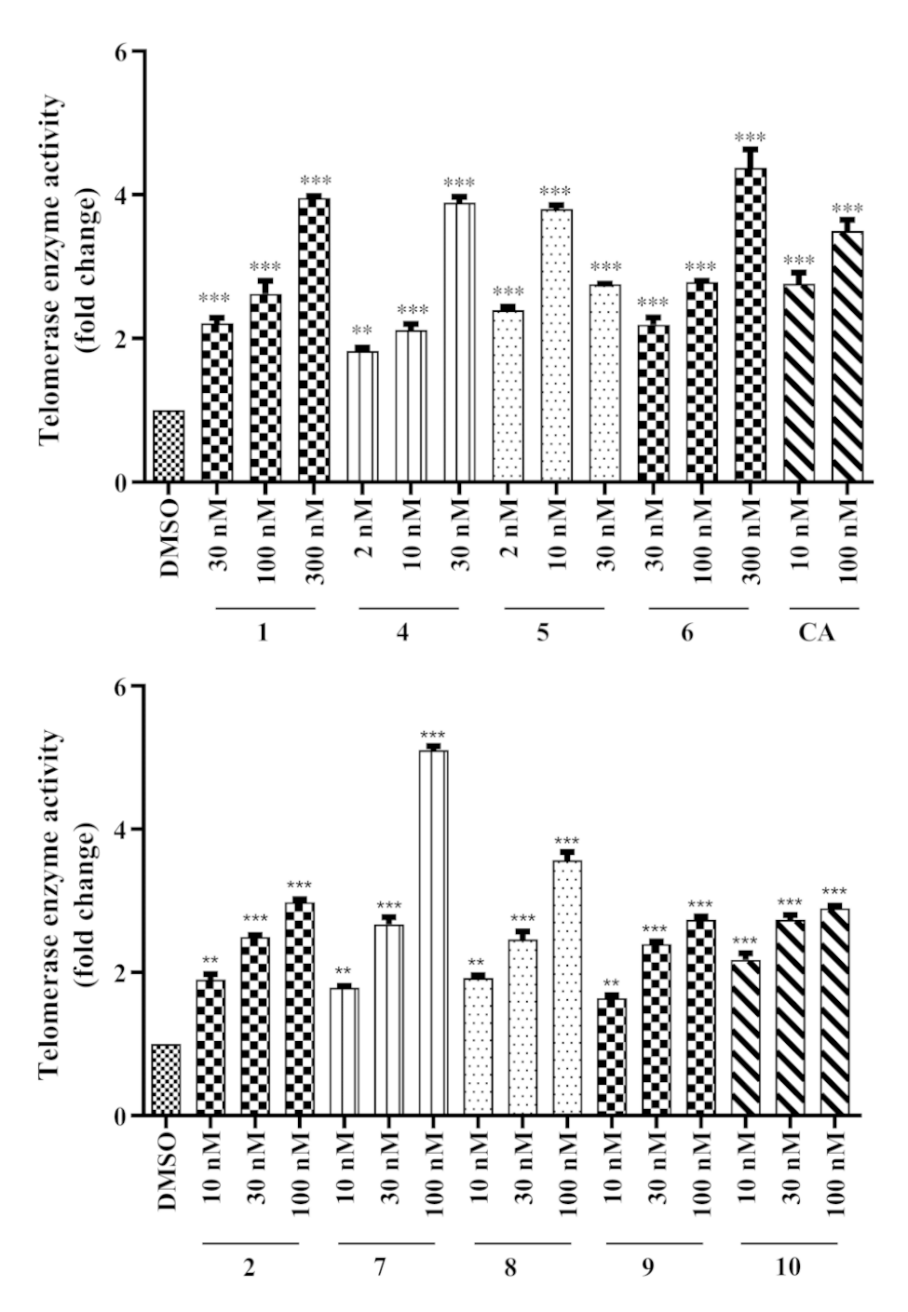
Telomerase activity of **1**, **2**, **4**–**10** in cell lysates measured by TeloTAGGG assay. Values are expressed as the fold change relative to the control DMSO. Error bars are presented as standard deviations (n = 2; *p ≤ 0.05, **p ≤ 0.001, ***p ≤ 0.005). CA: Cycloastragenol, positive control

Compared to the DMSO-treated control group, starting compounds and their derivatives exhibited promising telomerase activation (Fig. 4). While cyclocephagenol (**1**) increased telomerase enzyme activity at the highest concentration (300 nM) by 3.95-fold compared to DMSO, metabolites **4**, **5** and **6** improved enzyme activity as 3.89-fold (30 nM), 3.81-fold (10 nM) and 4.38-fold (300 nM), respectively. Additionally, the active dose of metabolite **2** (2.98-fold) and its derivatives **7** (5.11-fold), **8** (3.57-fold), **9** (2.74-fold), and **10** (2.90-fold) were 100 nM. The positive control, cycloastragenol, provided a 3.5-fold increase in telomerase activation at 100 nM (Fig. 4).

## Discussion

The fact that telomerase activators have the potential to be used not only in anti-aging therapies but also in regenerative medicine and degenerative diseases makes them very valuable [30–35, 42, 43]. One of the significant reluctancy to apply telomerase activators in the field has been their probable action on cellular transformation to initiate cancer. In recent years, studies on the discovery of telomerase activators and their molecular/mechanistic basis have increased with the demonstration that telomerase activation via small molecules does not cause cancer [44–49]. Several natural products have been reported as telomerase activators [50–52]. Among them, cycloastragenol plays a vital role in this field as the first commercialized compound [30–35, 42, 43]. Indeed, a recent finding by Deng et al. (2022) reporting that cycloastragenol inhibits tumor growth in vivo was also entirely meaningful for the field [53].

Based on the telomerase activator potentials of cycloartanes and our previous studies [37–39], we decided to carry out biotransformation studies on cyclocephagenol (**1**), a novel cycloartane-type sapogenin from *Astragalus* species, and its 12-hydroxy derivatives. As there was no bioactivity data on cyclocephagenol and its derivatives in terms of telomerase activation, we first evaluated the hTERT protein level of cyclocephagenol (**1**) and its 12-hydroxy derivatives (**2** and **3**) on HEKn cells. While cyclocephagenol (**1**) and **3** increased hTERT protein levels in a dose-dependent manner, metabolite **2** increased these protein levels at 10, 30, and 100 nM concentrations.

Since the hTERT protein levels of these compounds were notable, biotransformation studies were carried out on cyclocephagenol and its 12-hydroxy derivatives within the scope of this study. Biotransformation studies yielded seven new biotransformation products (Fig. 2). Structural elucidations were accomplished through 1D-, 2D-NMR, and HR-ESI-MS analysis. As in our previous study, *C. laburnicola* was found to catalyze oxidation, Baeyer-Villiger oxidation, ring opening, and dehydration reactions, respectively.

A biotransformation study on cyclocephagenol (**1**) resulted in the production of three compounds (**4**: 3(4)-seco, **5**: A ring-lacto, and **6**: 3-oxo), as in the biotransformation of cycloastragenol [37, 39]. Interestingly, in the biotransformation study performed with 12α-hydroxycyclocephagenol, a dehydration reaction (**10**) was also achieved in addition to these modifications (**7**: 3-oxidation; **8**: lactone formation; **9**: 3(4)-ring cleavage). This modification was also observed in the biotransformation of astragenol in our previous studies [37, 39].

In the continuation, biotransformation products were screened for their effects on telomerase enzyme activation, and Western Blot analysis was used for the preliminary screening. The effect of metabolites on hTERT protein level was investigated by Western Blot in the dose range of 2-300 nM. As a result, all metabolites were active compared to the DMSO-treated control group. The dose range for the TeloTAGGG study was determined as 2–30 nM for metabolites **4** and **5**, while the 30–300 nM range was chosen for **1** and **6**. Metabolites **2** and **7**–**10** were tested in the 10–100 nM dose range. Additionally, the most active doses of cycloastragenol (10 and 100 nM) were preferred as the positive control.

According to TeloTAGGG results, all molecules except metabolite **5** showed dose-dependent telomerase activation compared to the control. On the other hand, metabolite **5** showed a dose-dependent increase up to 10 nM and decreased at 30 nM. In addition, the effect of metabolites on hTERT protein level was consistent with TeloTAGGG results.

In evaluating the starting compounds (**1** and **2**), cyclocephagenol was found to be more active at a higher dose (3.96-fold at 300 nM). In comparison, metabolite **2** showed lower activity (2.98-fold) at 100 nM than cycloastragenol. The hydroxylation at C-12 caused a decrease in activity compared to our previous studies.

Metabolites **6** (4.38-fold at 300 nM) and **7** (5.11-fold at 100 nM), possessing a ketone functionality at C-3, are noteworthy because they are the most active derivatives. While hydroxylation at C-12 decreased telomerase activation in metabolite **2** (2.98-fold at 100 nM) compared to **1** (3.96-fold at 300 nM), a more active metabolite was obtained at a lower dose when oxidation in C-3 occurred in **2** (**7**). When examining the 3-oxo derivatives from a chemical point of view, (i) the hydrogen bond donor characteristic in C-3 has disappeared and has completely switched to the acceptor position, (ii) the polarity has decreased relatively compared to the starting compound, (iii) the chair conformation has changed in the A ring, resulting in a boat conformation. Although it is premature to infer a structure-activity relationship with a limited number of compounds, the copresence of oxidation at C-3 and monooxygenation at C-12 seems to be important in increasing telomerase activation.

Metabolites **5** and **8** are formed because of Baeyer-Villiger monooxygenase activity and contain seven-membered lactones in the A ring. Metabolite **5** provided a 3.81-fold telomerase activation at 10 nM, while metabolite **8** exhibited a 3.57-fold activation at a higher dose (100 nM). Contrary to 3-oxo derivatives, the hydroxylation at C-12 causes a decrease in activity in lactone derivatives. Lactone derivatives have similar properties with 3-oxo derivatives: (i) a high electron density at the hydrogen bond acceptor position in the A ring, (ii) partial boat conformation, (iii) slightly more polar character than 3-oxo derivatives.

After lactone ring formation, a lactone hydrolase enzyme catalyzes a further step to yield 3(4)-seco metabolites (metabolites **4**, **9**, and **10**). When these three molecules were tested, it was observed that metabolite **4** showed similar activity to metabolite **1** at lower doses, but metabolites **9** and **10** did not provide an increase in telomerase activation. Metabolites **4** and **9** carrying hydroxy at C-4 showed 3.89-fold activation at 30 nM and 2.74-fold at 100 nM, respectively; metabolite **10** containing C-4(28) double bonds provided a 2.9-fold activation at a concentration of 100 nM. As with lactone derivatives, hydroxylation at C-12 caused a decrease in activity. Considering the general physicochemical properties of 3(4)-seco, its more polar and ionizable nature due to the presence of a carboxylic acid is significant, especially for water solubility and possible electrostatic interactions. The carboxylic acid formed in the A ring (C-3) and the hydroxylation at C-4 forming an electron-rich region might be the key feature for molecular interactions with biological macromolecules (membrane/nuclear receptors and/or proteins) at the cellular level.

Collectively, the tested cyclocephagenol derivatives also demonstrated potent telomerase activation compared to the positive control cycloastragenol (varying between 1.02- and 1.46-fold).

## Conclusion

Based on the potent telomerase activity of *C. laburnicola* biotransformation metabolites deriving from cycloastragenol and astragenol, we made a further attempt to discover new activators. To be sure, cyclocephagenol and its 12-hydroxy derivatives were good candidates for telomerase activation, a preliminary test was performed on the hTERT protein level, revealing that both compounds were notably active. Thus, in continuation, biotransformation studies were initiated with *C. laburnicola* to give seven new molecules. As in our previous studies, *C. laburnicola* catalyzed oxidation at C-3 and ring-opening reactions by Baeyer-Villiger oxidation on the cyclocephagenol and 12α-hydroxycyclocephagenol. When the compounds were tested by TeloTAGGG assay, it was noteworthy that cyclocephagenol and its metabolites had more potency regarding telomerase activation than the positive control cycloastragenol.

## Experimental

### General experimental procedures

NMR spectra were obtained at 400 MHz for ^1^H and 100 MHz for ^13^C on a Varian Oxford AS400 spectrometer, and 600 MHz for ^1^H and 150 MHz for ^13^C on an Agilent 600 MHz Premium Compact in C_5_D_5_N with solvent peak used as a reference. Column chromatography (CC) was carried out on silica gel 60 (Merck 7734) and RP (C-18, Merck 9303). TLC analyses were carried out on Silica gel 60 F254 (Merck 5554) and RP-18 F254 (Merck) plates. Compounds were detected by UV and 20% aq. H_2_SO_4_ spraying reagent followed by heating at 105 °C for 1–2 min.

### Microorganism and starting compound

Cyclocephagenol (**1**) was donated by Bionorm Natural Products (İzmir, Türkiye). 12α-hydroxycyclocephagenol (**2**) and 12β-hydroxycyclocephagenol (**3**) was isolated in our previous study by using *A. eureka* [40]. *C. laburnicola* used in this study was isolated from fresh and healthy leaves of *A. angustifolius*. The original strain was deposited at the Bedir Laboratory with the deposit number 20131E4BL1 [39]. All cultures were maintained on potato dextrose agar (PDA, Merck, 1.10130.0500) slants and stored at 4 °C until use. Before biotransformation, the fungus was pre-cultivated on PDA in Petri dishes for 10 days at 25 °C.

### Microbial transformation procedure

Stock cultures stored at 4 °C in an agar slant were transferred to the fresh PDA medium and incubated at 25 °C for 10 days. Following incubation, Tween 80 (0.1%) was added to the fungi grown in the PDA medium, and a spore solution was obtained by scraping with an inoculation loop. The spore suspension was inoculated to potato dextrose broth (PDB, HKM Culture Media, 021053). After three days of incubation, the substrate (20 mg/mL in DMSO) was dosed at 1% (v/v) of the medium. Preparative-scale biotransformation studies were performed employing 100 mg of **1** for 4 days, 30 mg of **2** for 30 days, and 30 mg of **3** for 30 days with *C. laburnicola* (25 °C and 180 rpm).

### Extraction and isolation

After an incubation period, the fungal mycelia were filtered on a Buchner funnel, and the filtrate was extracted with EtOAc (×3). The organic phase was evaporated under reduced pressure to dryness.

Compounds **4**–**6** were isolated from the EtOAc extract (120 mg) of *C. laburnicola* with **1**. This extract was subjected to vacuum-liquid chromatography (VLC) loaded with reversed-phase silica gel (RP-C18, 30 g) to yield **4** (22.6 mg), **6** (9.2 mg), and one impure fraction (A) after elution with an ACN:H_2_O gradient (40:60, 50:50, 60:40, 100:0). Fraction A (7.9 mg) was chromatographed on a silica gel column (10 g) using CHCl_3_:MeOH gradient (100:0, 98:2, 97:3) to afford 29 fractions. Fractions from A22 to A26 (6.8 mg) were combined and re-chromatographed over a silica gel column (10 g) using CHCl_3_:IPA gradient (99:1, 97:3) to afford **5** (2.3 mg).

Compounds **7**–**10** were isolated from the EtOAc extract of **2** (241 mg) by *C. laburnicola*. This extract was chromatographed on a silica gel column (33 g) using *n*-hexane:EtOAc:MeOH:H_2_O (10:10:2:0, 10:10:3:0, 0:100:17.5:13.5) gradient to give four impure fractions (Fractions A-D). Each impure fraction was subjected to vacuum-liquid chromatography (VLC) loaded with reversed-phase silica gel (RP-C18, 20 g) to yield **7** (7.2 mg), **8** (3.7 mg), **9** (3.4 mg), and **10** (2 mg).

### Spectral data of 4–10

**Metabolite 4**: ^1^H-NMR (C_5_D_5_N, 400 MHz) and ^13^C-NMR (C_5_D_5_N, 100 MHz): see Tables 1 and 2; HR-ESI-MS (positive ion mode): *m/z* 545.34691 (C_30_H_50_NaO_7_, calcd. 545.34487).

**Table 1:** ^1^H NMR data of compounds **4**–**10** (in C_5_D_5_N)

		4^a^		5^a^		6^a^		7^b^		8^a^		9^b^		10^b^	
**H**		δ_H_ (*J* in Hz)		δ_H_ (*J* in Hz)		δ_H_ (*J* in Hz)		δ_H_ (*J* in Hz)		δ_H_ (*J* in Hz)		δ_H_ (*J* in Hz)		δ_H_ (*J* in Hz)	
**1**		1.67 m, 2.71 m		1.28 m, 1.95 m		1.25 m, 1.96 m		1.38 m, 2.11 m		1.42 m, 1.99 m		1.82 m, 2.77 m		1.80 t (13.1), 2.53 m	
**2**		2.60 m, 2.96 m		2.60 m, 2.88 m		2.43 ddd (14.3, 8.1, 6.8), 2.70 ddd (14.2, 8.6, 5.9)		2.43 ddd (14.1, 8.4, 6.0), 2.77 m		2.58 dd (13.2, 5.1), 2.89 td (13.3, 7.0)		2.74 m, 3.00 m		2.70 m, 2.95 m	
**3**		-		-		-		-		-		-		-	
**4**		-		-		-		-		-		-		-	
**5**		2.37 d (8.4)		2.40 d (9.3)		2.18 d (9.7)		2.29 d (9.8)		2.44 d (9.3)		2.45 d (8.3)		2.78 d (9.7)	
**6**		4.05 dd (12.7, 5.6)		3.66 m		3.64 m		3.70 dd (9.9, 4.9)		3.70 m		4.10 m		3.64 m	
**7**		1.58 m, 1.79 m		1.51 m, 1.69 m		1.58 m, 1.71 m		1.75 (2 H)		1.62 m, 1.70 m		1.72 m, 1.85 m		1.65 m, 1.90 m	
**8**		1.59 m		1.72 m		1.86 m		1.88 m		1.72 m		1.67 m		1.94 m	
**9**		-		-		-		-		-		-		-	
**10**		-		-		-		-		-		-		-	
**11**		1.15 m, 2.30 m		0.80 m, 2.02 m		0.91 m, 1.98 m		1.76 m, 2.38 m		1.67 m, 2.35 dd (14.6, 5.8)		2.04 dd (14.5, 8.4), 2.69 dd (14.5, 6.8)		2.11 m, 2.73 m	
**12**		1.68 m, 1.84 m		1.74 m, 1.85 m		1.69 m, 1.82 m		4.26 ddd (9.2, 6.1, 2.6)		4.25 t (7.6)		4.28 t (7.9)		4.28 t (7.7)	
**13**		-		-		-		-		-		-		-	
**14**		-		-		-		-		-		-		-	
**15**		1.77 m, 2.15 m		1.80 m, 2.15 m		1.79 m, 2.17 m		1.90 m, 2.19 dd (12.7, 7.9)		1.89 m, 2.21 dd (12.5, 7.8)		1.89 m, 2.23 dd (12.6, 7.7)		1.91 m, 2.21 dd (12.6, 7.9)	
**16**		4.89 dd (13.4, 7.7)		4.68 dt (8.2, 4.2)		4.90 tdd (8.1, 5.4, 3.2)		4.93 m		4.93 m		4.93 q (7.2)		4.94 m	
**17**		2.06 d (7.7)		2.10 d (7.7)		2.07 d (7.3)		2.97 d (8.0)		2.96 d (8.1)		2.97 d (8.3)		2.99 d (8.0)	
**18**		1.71 s		1.70 s		1.68 s		1.71 s		1.70 s		1.76 s		1.75 s	
**19**		0.59 d (4.4), 1.00 d (4.4)		0.53 d (5.0), 0.79 d (4.9)		0.31 d (4.1), 0.64 d (4.2)		0.48 d (4.2), 0.70 d (4.2)		0.67 d (4.8), 0.84 d (4.9)		0.74 d (4.4), 1.06 d (4.4)		0.63 d (4.4), 0.73 d (4.4)	
**20**		-		-		-		-		-		-		-	
**21**		1.55 s		1.60 s		1.57 s		1.99 s		1.97 s		1.98 s		1.99 s	
**22**		1.24 m, 3.03 m		1.26 m, 3.11 m		1.24 m, 3.08 td (13.5, 4.5)		2.13 dd (13.1, 3.7)		2.10 d (13.4), 2.78 t (12.2)		2.13 m, 2.78 m		2.13 m, 2.81 m	
**23**		1.87 m, 2.16 m		1.91 m, 2.21 m		1.87 m, 2.19 m		1.94 dd (14.0, 3.7), 2.34 t (3.4)		1.95 m, 2.35 m		1.93 m, 2.34 t (13.9)		1.94 m, 2.34 t (14.0)	
**24**		3.66 brs		3.70 brs		3.66 brs		3.73 brs		3.72 brs		3.73 brs		3.74 s	
**25**		-		-		-		-		-		-		-	
**26**		1.44 s		1.47 s		1.44 s		1.57 s		1.56 s		1.58 s		1.59 s	
**27**		1.27 s		1.32 s		1.28 s		1.39 s		1.39 s		1.40 s		1.40 s	
**28**		1.76 s		1.93 s		1.74 s		1.78 s		1.94 s		1.80 s		5.09 s, 5.21 s	
**29**		1.60 s		1.67 s		1.42 s		1.50 s		1.70 s		1.65 s		1.89 s	
**30**		0.94 s		0.94 s		0.92 s		1.28 s		1.27 s		1.31 s		1.33 s	

^a^400 MHz, ^b^600 MHz, †Overlapped signals

**Table 2:** ^13^C NMR data of compounds **4**–**10** (in C_5_D_5_N)

	4^a^	5^a^	6^a^	7^b^	8^a^	9^b^	10^b^
**C**	δ_C_	δ_C_	δ_C_	δ_C_	δ_C_	δ_C_	δ_C_
**1**	31.9†	33.0	31.6	32.3	32.5	33.0	31.1
**2**	32.9	34.3	35.6	36.0	33.6	34.0	33.1
**3**	176.0	174.5	216.6	217.4	173.6	179.5	177.3
**4**	75.7	86.1	50.3	51.0	85.3	76.4	147.1
**5**	53.3	53.8	53.3	54.0	53.2	54.2	55.7
**6**	71.1	71.6	69.0	70.0	70.9	71.9	69.3
**7**	36.9	37.9	38.1	39.1	37.4	37.7	36.7
**8**	46.8†	48.6	47.8	49.1	48.1	47.8	49.0
**9**	25.0	22.3	20.9	21.8	21.7	29.2	30.7
**10**	28.5	26.5	28.1	28.7	25.8	25.7	22.2
**11**	26.5	27.1	26.0	38.6	38.2	38.7	39.0
**12**	34.2	34.6	33.8	74.5	73.0	74.0	73.4
**13**	46.9†	47.1	46.5	46.8	51.0	47.2	47.0
**14**	45.3	46.1	45.7	52.0	46.0	51.8	51.8
**15**	48.7	49.3	48.1	51.4	51.0	51.9	51.3
**16**	73.8	74.4	73.7	72.5	71.9	72.7	72.3
**17**	60.9	61.5	60.7	55.7	54.9	55.7	55.5
**18**	22.0	22.5	21.5	21.2	20.5	21.5	21.2
**19**	31.9†	31.8	30.8	31.4	30.8	32.3	32.4
**20**	78.8	79.5	78.8	79.9	79.3	80.0	79.8
**21**	28.5	29.1	28.4	27.8	27.1	27.8	27.7
**22**	26.4	27.1	26.4	29.4	28.6	29.2	29.1
**23**	23.8	24.5	23.8	24.3	23.6	24.3	24.2
**24**	68.4	69.1	68.4	69.2	68.5	69.3	69.1
**25**	75.1	75.9	75.1	76.3	75.5	76.4	76.2
**26**	28.4	29.1	28.4	29.1	28.4	29.1	28.9
**27**	27.8	28.6	27.8	28.7	28.0	28.8	28.6
**28**	33.3	32.4	28.4	29.3	31.7	34.0	115.3
**29**	28.8	28.5	20.1	20.7	27.8	29.5	20.7
**30**	20.7	21.5	20.4	22.6	22.1	22.6	22.3

^a^100 MHz, ^b^150 MHz, †Overlapped signals

**Metabolite 5**: ^1^H-NMR (C_5_D_5_N, 400 MHz) and ^13^C-NMR (C_5_D_5_N, 100 MHz): see Tables 1 and 2; HR-ESI-MS (positive ion mode): *m/z* 527.33635 (C_30_H_48_NaO_6_, calcd. 527.33431).

**Metabolite 6**: ^1^H-NMR (C_5_D_5_N, 400 MHz) and ^13^C-NMR (C_5_D_5_N, 100 MHz): see Tables 1 and 2; HR-ESI-MS (positive ion mode): *m/z* 511.33940 (C_30_H_48_NaO_5_, calcd. 511.33939).

**Metabolite 7**: ^1^H-NMR (C_5_D_5_N, 600 MHz) and ^13^C-NMR (C_5_D_5_N, 150 MHz): see Tables 1 and 2; HR-ESI-MS (negative ion mode): *m/z* 503.33835 (C_30_H_47_O_6_, calcd. 503.33781).

**Metabolite 8**: ^1^H-NMR (C_5_D_5_N, 400 MHz) and ^13^C-NMR (C_5_D_5_N, 100 MHz): see Tables 1 and 2; HR-ESI-MS (negative ion mode): *m/z* 519.33289 (C_30_H_47_O_7_, calcd. 519.33272).

**Metabolite 9**: ^1^H-NMR (C_5_D_5_N, 600 MHz) and ^13^C-NMR (C_5_D_5_N, 150 MHz): see Tables 1 and 2; HR-ESI-MS (negative ion mode): *m/z* 537.34344 (C_30_H_49_O_8_, calcd. 537.34329).

**Metabolite 10**: ^1^H-NMR (C_5_D_5_N, 600 MHz) and ^13^C-NMR (C_5_D_5_N, 150 MHz): see Tables 1 and 2; HR-ESI-MS (negative ion mode): *m/z* 519.33122 (C_30_H_47_O_7_, calcd. 519.33272).

**Bioactivity studies**

**Cell line and culture conditions**

Primary human epidermal keratinocyte cells (HEKn) (ATCC; PCS-200-010) were cultured in Dermal Cell Basal Media (ATCC; PCS-200-030) supplemented with Keratinocyte Growth Kit (ATCC; PCS-200-040) according to the manufacturer’s instructions at 37 °C under humidified 5% CO_2_. HEKn cells were seeded at a density of 2500 to 5000 cells per cm^2^ when they reached 70–80% confluency.

### Immunoblotting studies

The lysates of cells were prepared in RIPA buffer (1% Nonidet P-40, 0.5% sodium deoxycholate, and 0.1% SDS in 1X PBS, pH 8.0) with a protease inhibitor cocktail (Roche, Switzerland). Protein concentrations were determined via BCA assay (Thermo Fisher Scientific, US). At first, equal amounts of prepared were denatured with 4X Laemmli buffer at 95 °C. Then denatured samples were loaded into gels and separated by SDS-PAGE electrophoresis. After electrophoresis was completed, gels were transferred to PVDF membranes (EMD Millipore, US, IPVH00010). After blocking membranes in 5% non-fat dry milk prepared in 1X PBS-0.1% Tween-20, membranes were labeled with primary and secondary antibodies. In this study, antibodies against hTERT (Origene, TA301588) and GAPDH (CST, 5174) were used. Secondary antibodies (Goat anti-rabbit-31460 and Goat anti-mouse-31430) were used. The proteins’ chemiluminescence signals were determined with Clarity ECL substrate solution (BIORAD, US, 1705061) by Vilber Loumart FX-7 (Thermo Fisher Scientific, US). ImageJ software (http://imagej.nih.gov/ij/) was used for Western blot images.

### Telomerase activity assay

The identification of telomerase enzyme activity was performed in HEKn cells by TeloTAGGG Telomerase PCR Plus Kit (Sigma Aldrich, 12013789001) according to the supplier’s instruction. The obtained data were presented as a fold change of DMSO used as a solvent control. Experiments were done in two biological experiments with two technical replicates. A one-way ANOVA Post Hoc test was used for statistical analysis of this assay using GraphPad Prism software. The significance of the differences was determined as *p ≤ 0.05, **p ≤ 0.001, ***p ≤ 0.005.

## Electronic supplementary material

Below is the link to the electronic supplementary material.

**Additional file 1.** Mass and NMR spectra of biotransformation products **4-10.**

## Data Availability

All data generated and analyzed in this study are included in this article and Additional files.
