# Supplementary material for: Potent telomerase activators from a novel sapogenin via biotransformation utilizing Camarosporium laburnicola, an endophytic fungus
Source: Microb Cell Fact. 2023 Apr 6;22:66. doi: 10.1186/s12934-023-02069-3 (PMC10080871; doi:10.1186/s12934-023-02069-3)
Supplement: Supplementary file 1 — Additional file 1. Mass and NMR spectra of biotransformation products 4-10. [file 12934_2023_2069_MOESM1_ESM.docx]

**SUPPLEMENTARY INFORMATION**

**BIOCATALYSIS OF CYCLOCEPHAGENOL AND ITS DERIVATIVES BY *CAMAROSPORIUM LABURNICOLA* TO OBTAIN TELOMERASE ACTIVATORS**

Melis Küçüksolak^1^, Sinem Yılmaz^2,3^, Petek Ballar-Kırmızıbayrak^4^, Erdal Bedir^1*^

^1^Department of Bioengineering, Izmir Institute of Technology, 35430, Urla, İzmir, Türkiye

^2^Department of Biotechnology, Graduate School of Natural and Applied Sciences, Ege University, Bornova, İzmir, Türkiye

^3^Department of Bioengineering, Faculty of Engineering, University of Alanya Aladdin Keykubat, Antalya, Türkiye

^4^Department of Biochemistry, Faculty of Pharmacy, Ege University, 35100, Bornova, İzmir, Türkiye

***Corresponding Author:**

**Erdal BEDİR:** İzmir Institute of Technology, 35430 Urla/İzmir, Türkiye; phone: +90(232) 750 6952; email: erdalbedir@iyte.edu.tr

**LIST OF FIGURES**

[Fig. S 1. Chemical structure of compound **4**. 4](#_Toc129899727)

[Fig. S 2. HR-ESI-MS spectrum of compound **4**. 4](#_Toc129899728)

[Fig. S 3. ^1^H-NMR spectrum of compound **4**. 5](file:///\\Mac\Home\Desktop\Camarosporium%20yayın\Microbial%20Cell%20Factories\Supplementary%20Information%20-%20Copy.docx#_Toc129899729)

[Fig. S 4. ^13^C-NMR spectrum of compound **4**. 6](file:///\\Mac\Home\Desktop\Camarosporium%20yayın\Microbial%20Cell%20Factories\Supplementary%20Information%20-%20Copy.docx#_Toc129899730)

[Fig. S 5. ^1^H-^1^H COSY spectrum of compound **4**. 7](file:///\\Mac\Home\Desktop\Camarosporium%20yayın\Microbial%20Cell%20Factories\Supplementary%20Information%20-%20Copy.docx#_Toc129899731)

[Fig. S 6. HSQC spectrum of compound **4**. 8](file:///\\Mac\Home\Desktop\Camarosporium%20yayın\Microbial%20Cell%20Factories\Supplementary%20Information%20-%20Copy.docx#_Toc129899732)

[Fig. S 7. HMBC spectrum of compound **4**. 9](file:///\\Mac\Home\Desktop\Camarosporium%20yayın\Microbial%20Cell%20Factories\Supplementary%20Information%20-%20Copy.docx#_Toc129899733)

[Fig. S 8. Chemical structure of compound **5**. 10](#_Toc129899734)

[Fig. S 9. HR-ESI-MS spectrum of compound **5**. 10](#_Toc129899735)

[Fig. S 10. ^1^H-NMR spectrum of compound **5**. 11](file:///\\Mac\Home\Desktop\Camarosporium%20yayın\Microbial%20Cell%20Factories\Supplementary%20Information%20-%20Copy.docx#_Toc129899736)

[Fig. S 11. ^13^C-NMR spectrum of compound **5**. 12](file:///\\Mac\Home\Desktop\Camarosporium%20yayın\Microbial%20Cell%20Factories\Supplementary%20Information%20-%20Copy.docx#_Toc129899737)

[Fig. S 12. ^1^H-^1^H COSY spectrum of compound **5**. 13](file:///\\Mac\Home\Desktop\Camarosporium%20yayın\Microbial%20Cell%20Factories\Supplementary%20Information%20-%20Copy.docx#_Toc129899738)

[Fig. S 13. HSQC spectrum of compound **5**. 14](file:///\\Mac\Home\Desktop\Camarosporium%20yayın\Microbial%20Cell%20Factories\Supplementary%20Information%20-%20Copy.docx#_Toc129899739)

[Fig. S 14. HMBC spectrum of compound **5**. 15](file:///\\Mac\Home\Desktop\Camarosporium%20yayın\Microbial%20Cell%20Factories\Supplementary%20Information%20-%20Copy.docx#_Toc129899740)

[Fig. S 15. Chemical structure of compound **6**. 16](#_Toc129899741)

[Fig. S 16. HR-ESI-MS spectrum of compound **6**. 16](#_Toc129899742)

[Fig. S 17. ^1^H-NMR spectrum of compound **6**. 17](file:///\\Mac\Home\Desktop\Camarosporium%20yayın\Microbial%20Cell%20Factories\Supplementary%20Information%20-%20Copy.docx#_Toc129899743)

[Fig. S 18. ^13^C-NMR spectrum of compound **6**. 18](file:///\\Mac\Home\Desktop\Camarosporium%20yayın\Microbial%20Cell%20Factories\Supplementary%20Information%20-%20Copy.docx#_Toc129899744)

[Fig. S 19. ^1^H-^1^H COSY spectrum of compound **6**. 19](file:///\\Mac\Home\Desktop\Camarosporium%20yayın\Microbial%20Cell%20Factories\Supplementary%20Information%20-%20Copy.docx#_Toc129899745)

[Fig. S 20. HSQC spectrum of compound **6**. 20](file:///\\Mac\Home\Desktop\Camarosporium%20yayın\Microbial%20Cell%20Factories\Supplementary%20Information%20-%20Copy.docx#_Toc129899746)

[Fig. S 21. HMBC spectrum of compound **6**. 21](file:///\\Mac\Home\Desktop\Camarosporium%20yayın\Microbial%20Cell%20Factories\Supplementary%20Information%20-%20Copy.docx#_Toc129899747)

[Fig. S 22. Chemical structure of compound **7**. 22](#_Toc129899748)

[Fig. S 23. HR-ESI-MS spectrum of compound **7**. 22](#_Toc129899749)

[Fig. S 24. ^1^H-NMR spectrum of compound **7**. 23](file:///\\Mac\Home\Desktop\Camarosporium%20yayın\Microbial%20Cell%20Factories\Supplementary%20Information%20-%20Copy.docx#_Toc129899750)

[Fig. S 25. ^13^C-NMR spectrum of compound **7**. 24](file:///\\Mac\Home\Desktop\Camarosporium%20yayın\Microbial%20Cell%20Factories\Supplementary%20Information%20-%20Copy.docx#_Toc129899751)

[Fig. S 26. ^1^H-^1^H COSY spectrum of compound **7**. 25](file:///\\Mac\Home\Desktop\Camarosporium%20yayın\Microbial%20Cell%20Factories\Supplementary%20Information%20-%20Copy.docx#_Toc129899752)

[Fig. S 27. HSQC spectrum of compound **7**. 26](file:///\\Mac\Home\Desktop\Camarosporium%20yayın\Microbial%20Cell%20Factories\Supplementary%20Information%20-%20Copy.docx#_Toc129899753)

[Fig. S 28. HMBC spectrum of compound **7**. 27](file:///\\Mac\Home\Desktop\Camarosporium%20yayın\Microbial%20Cell%20Factories\Supplementary%20Information%20-%20Copy.docx#_Toc129899754)

[Fig. S 29. Chemical structure of compound **8**. 28](#_Toc129899755)

[Fig. S 30. HR-ESI-MS spectrum of compound **8**. 28](#_Toc129899756)

[Fig. S 31. ^1^H-NMR spectrum of compound **8**. 29](file:///\\Mac\Home\Desktop\Camarosporium%20yayın\Microbial%20Cell%20Factories\Supplementary%20Information%20-%20Copy.docx#_Toc129899757)

[Fig. S 32. ^13^C-NMR spectrum of compound **8**. 30](file:///\\Mac\Home\Desktop\Camarosporium%20yayın\Microbial%20Cell%20Factories\Supplementary%20Information%20-%20Copy.docx#_Toc129899758)

[Fig. S 33. ^1^H-^1^H COSY spectrum of compound **8**. 31](file:///\\Mac\Home\Desktop\Camarosporium%20yayın\Microbial%20Cell%20Factories\Supplementary%20Information%20-%20Copy.docx#_Toc129899759)

[Fig. S 34. HSQC spectrum of compound **8**. 32](file:///\\Mac\Home\Desktop\Camarosporium%20yayın\Microbial%20Cell%20Factories\Supplementary%20Information%20-%20Copy.docx#_Toc129899760)

[Fig. S 35. HMBC spectrum of compound **8**. 33](file:///\\Mac\Home\Desktop\Camarosporium%20yayın\Microbial%20Cell%20Factories\Supplementary%20Information%20-%20Copy.docx#_Toc129899761)

[Fig. S 36. Chemical structure of compound **9**. 34](#_Toc129899762)

[Fig. S 37. HR-ESI-MS spectrum of compound **9**. 34](#_Toc129899763)

[Fig. S 38. ^1^H-NMR spectrum of compound **9**. 35](file:///\\Mac\Home\Desktop\Camarosporium%20yayın\Microbial%20Cell%20Factories\Supplementary%20Information%20-%20Copy.docx#_Toc129899764)

[Fig. S 39. ^13^C-NMR spectrum of compound **9**. 36](file:///\\Mac\Home\Desktop\Camarosporium%20yayın\Microbial%20Cell%20Factories\Supplementary%20Information%20-%20Copy.docx#_Toc129899765)

[Fig. S 40. ^1^H-^1^H COSY spectrum of compound **9**. 37](file:///\\Mac\Home\Desktop\Camarosporium%20yayın\Microbial%20Cell%20Factories\Supplementary%20Information%20-%20Copy.docx#_Toc129899766)

[Fig. S 41. HSQC spectrum of compound **9**. 38](file:///\\Mac\Home\Desktop\Camarosporium%20yayın\Microbial%20Cell%20Factories\Supplementary%20Information%20-%20Copy.docx#_Toc129899767)

[Fig. S 42. HMBC spectrum of compound **9**. 39](file:///\\Mac\Home\Desktop\Camarosporium%20yayın\Microbial%20Cell%20Factories\Supplementary%20Information%20-%20Copy.docx#_Toc129899768)

[Fig. S 43. Chemical structure of compound **10**. 40](#_Toc129899769)

[Fig. S 44. HR-ESI-MS spectrum of compound **10**. 40](#_Toc129899770)

[Fig. S 45. ^1^H-NMR spectrum of compound **10**. 41](file:///\\Mac\Home\Desktop\Camarosporium%20yayın\Microbial%20Cell%20Factories\Supplementary%20Information%20-%20Copy.docx#_Toc129899771)

[Fig. S 46. ^13^C-NMR spectrum of compound **10**. 42](file:///\\Mac\Home\Desktop\Camarosporium%20yayın\Microbial%20Cell%20Factories\Supplementary%20Information%20-%20Copy.docx#_Toc129899772)

[Fig. S 47. ^1^H-^1^H COSY spectrum of compound **10**. 43](file:///\\Mac\Home\Desktop\Camarosporium%20yayın\Microbial%20Cell%20Factories\Supplementary%20Information%20-%20Copy.docx#_Toc129899773)

[Fig. S 48. HSQC spectrum of compound **10**. 44](file:///\\Mac\Home\Desktop\Camarosporium%20yayın\Microbial%20Cell%20Factories\Supplementary%20Information%20-%20Copy.docx#_Toc129899774)

[Fig. S 49. HMBC spectrum of compound **10**. 45](file:///\\Mac\Home\Desktop\Camarosporium%20yayın\Microbial%20Cell%20Factories\Supplementary%20Information%20-%20Copy.docx#_Toc129899775)

Fig. S 1. Chemical structure of compound **4**.

**
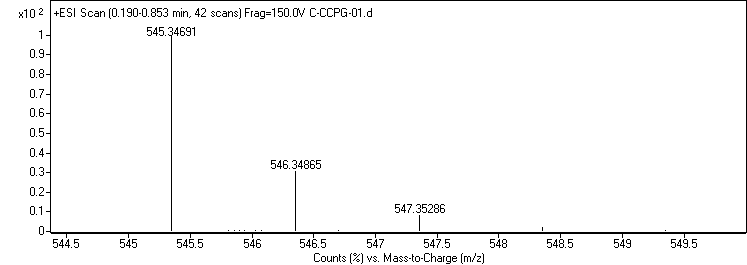
**

**[M+Na]^+^**

Fig. S 2. HR-ESI-MS spectrum of compound **4**.


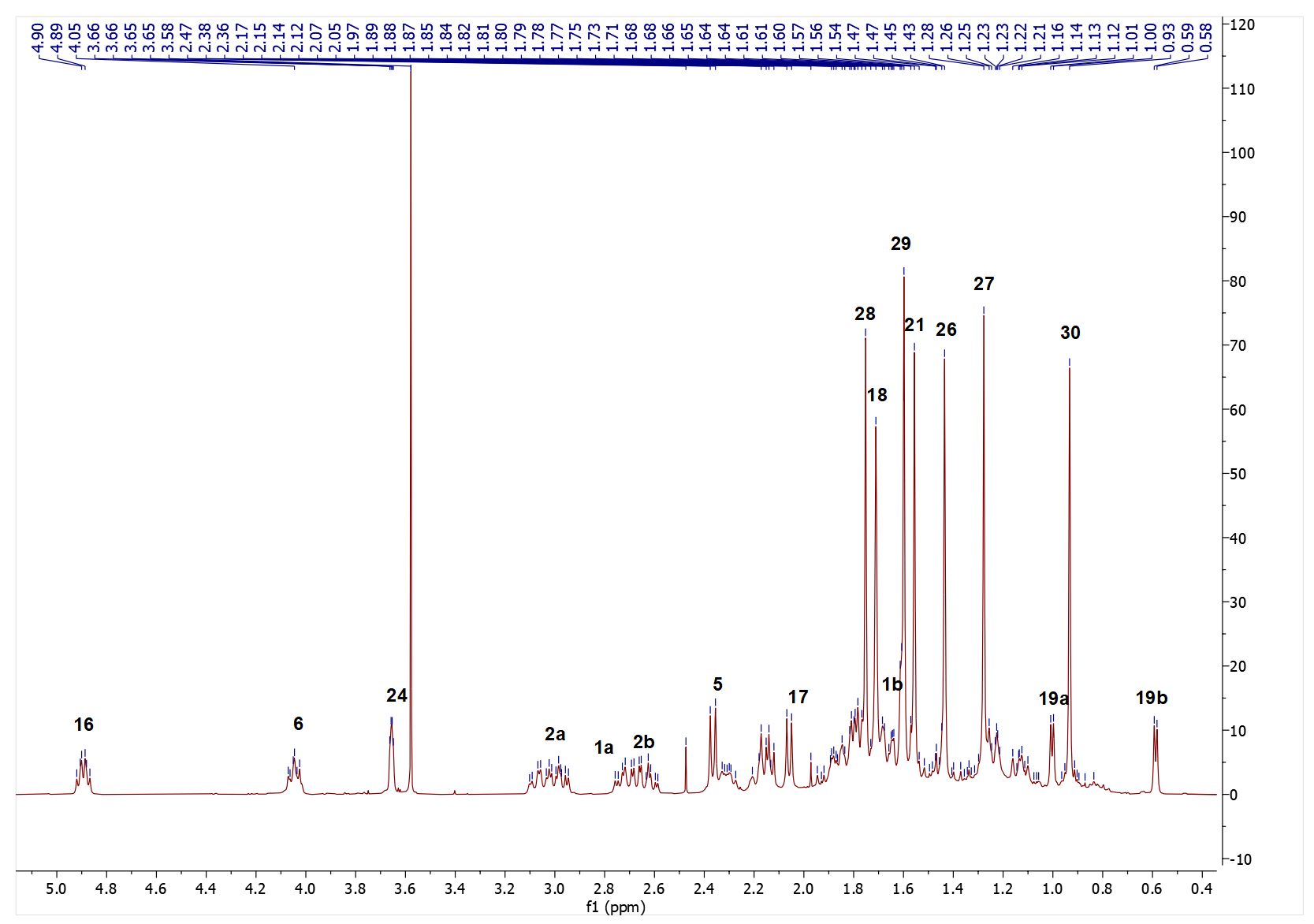


Fig. S 3. ^1^H-NMR spectrum of compound **4**.


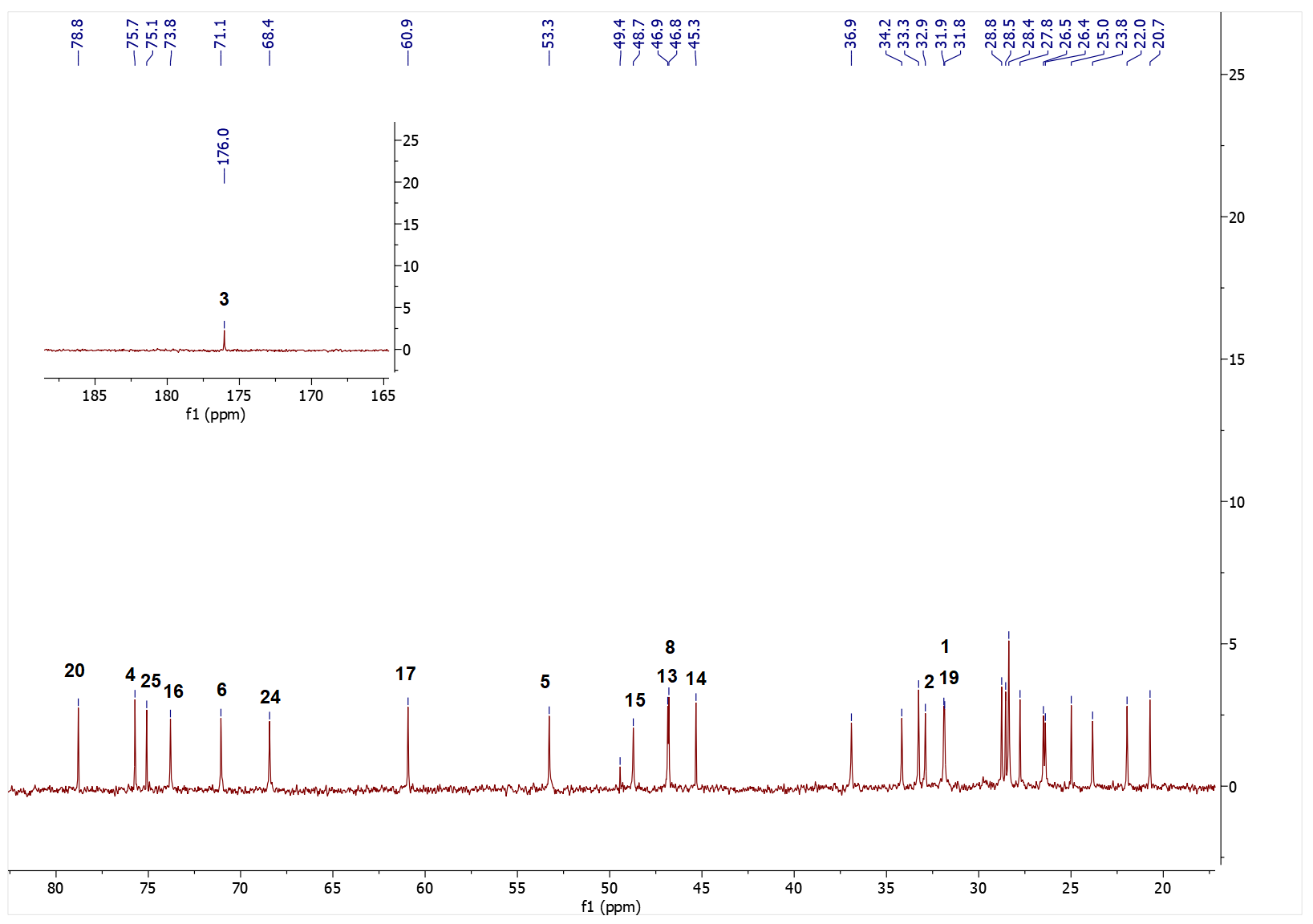


Fig. S 4. ^13^C-NMR spectrum of compound **4**.


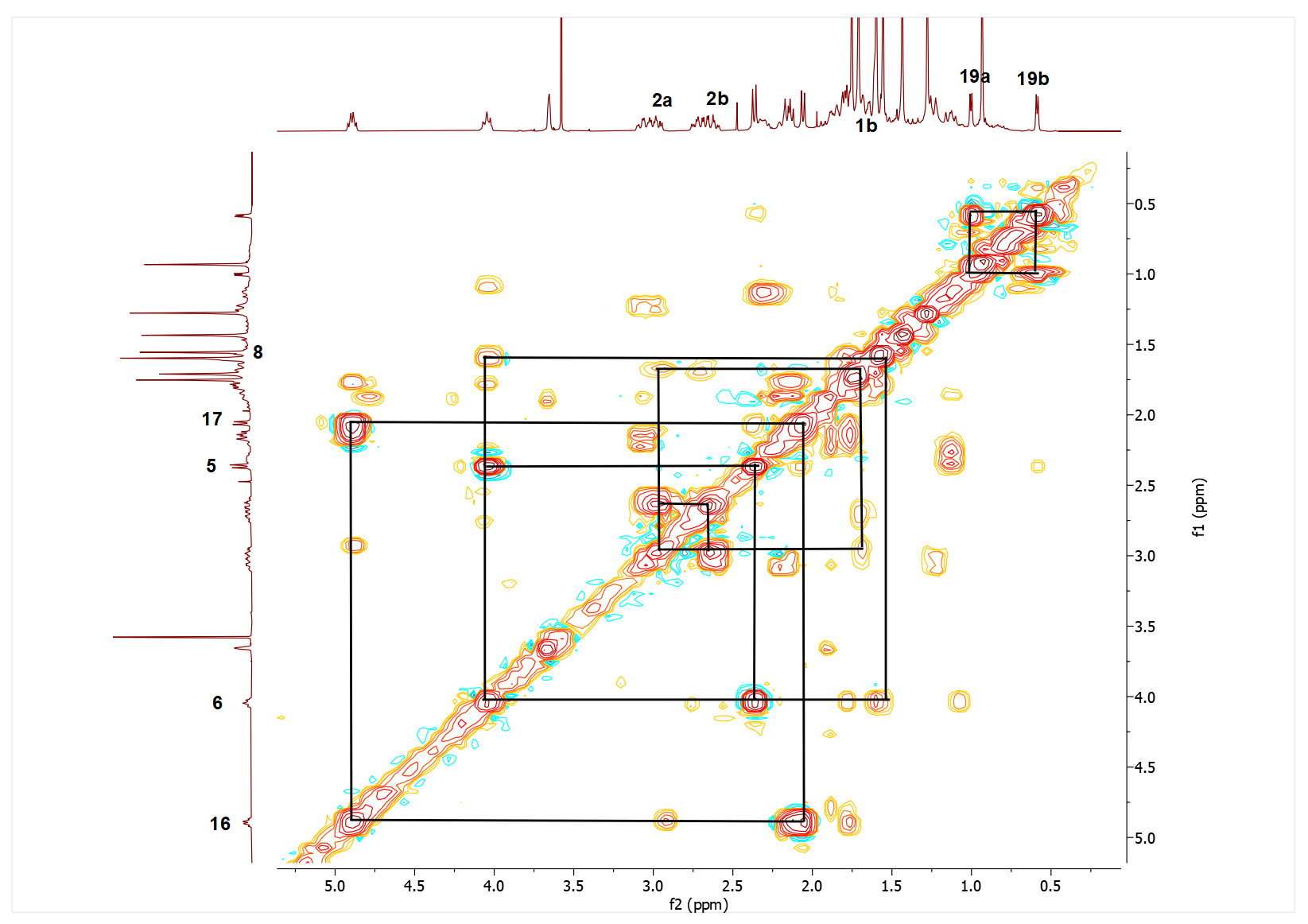


Fig. S 5. ^1^H-^1^H COSY spectrum of compound **4**.


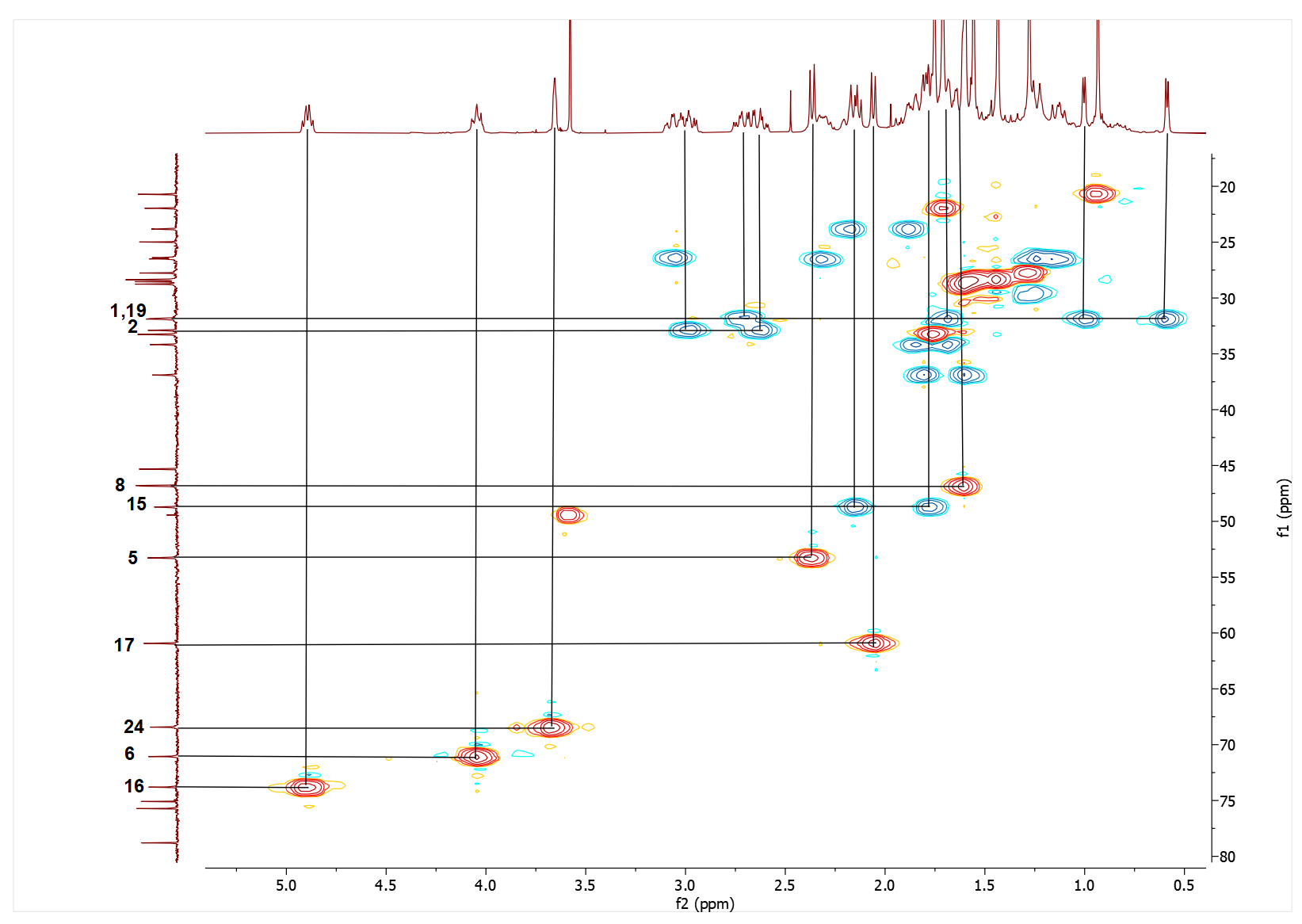


Fig. S 6. HSQC spectrum of compound **4**.


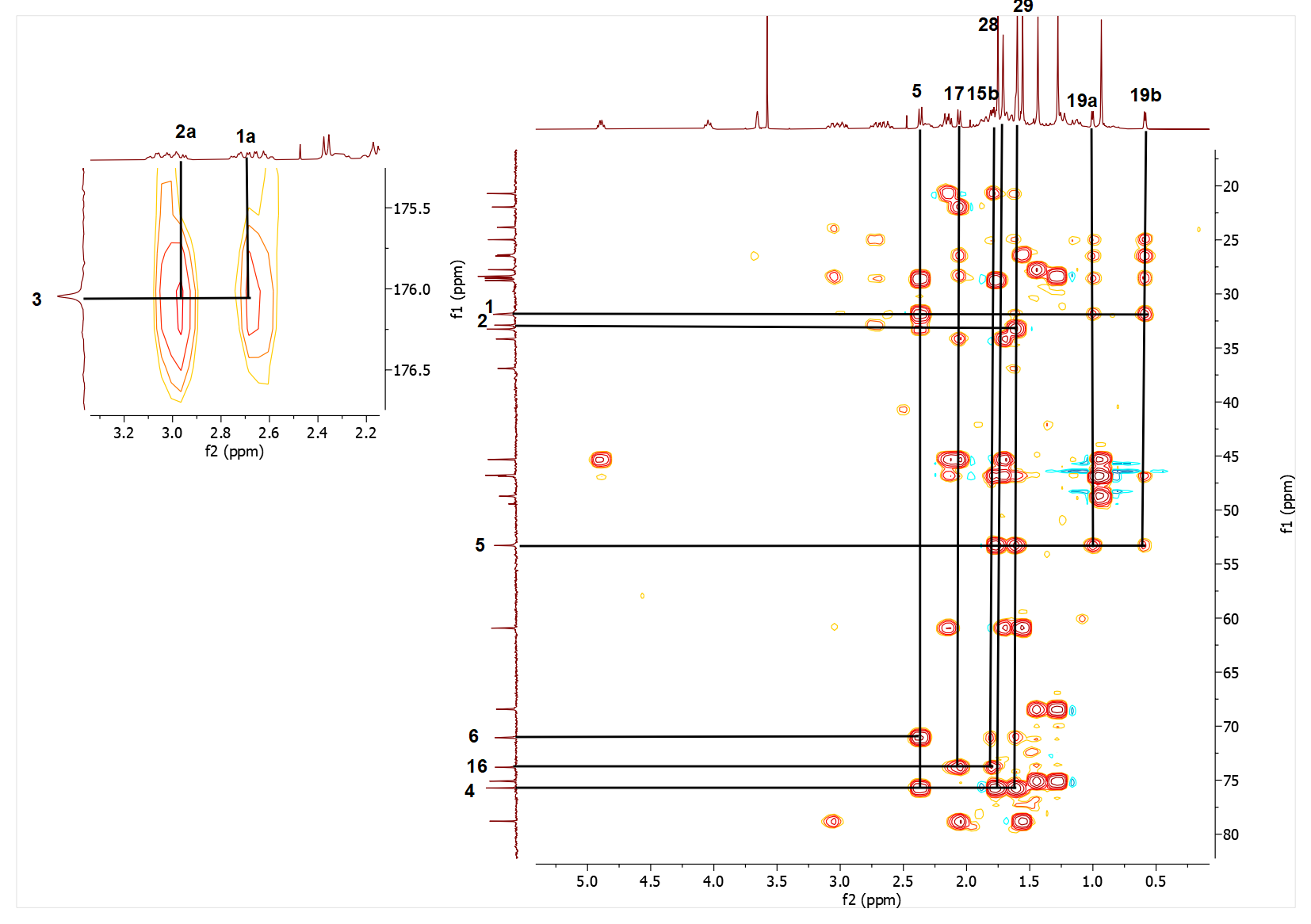

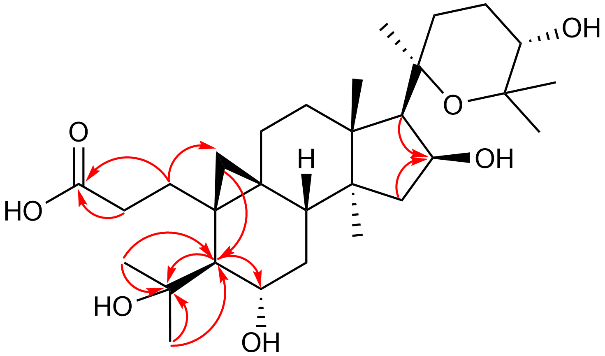


Fig. S 7. HMBC spectrum of compound **4**.

Fig. S 8. Chemical structure of compound **5**.


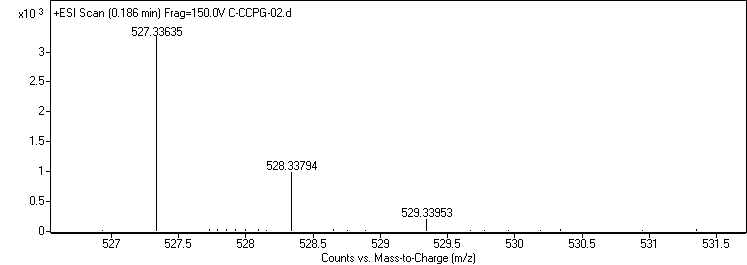


**[M+Na]^+^**

Fig. S 9. HR-ESI-MS spectrum of compound **5**.


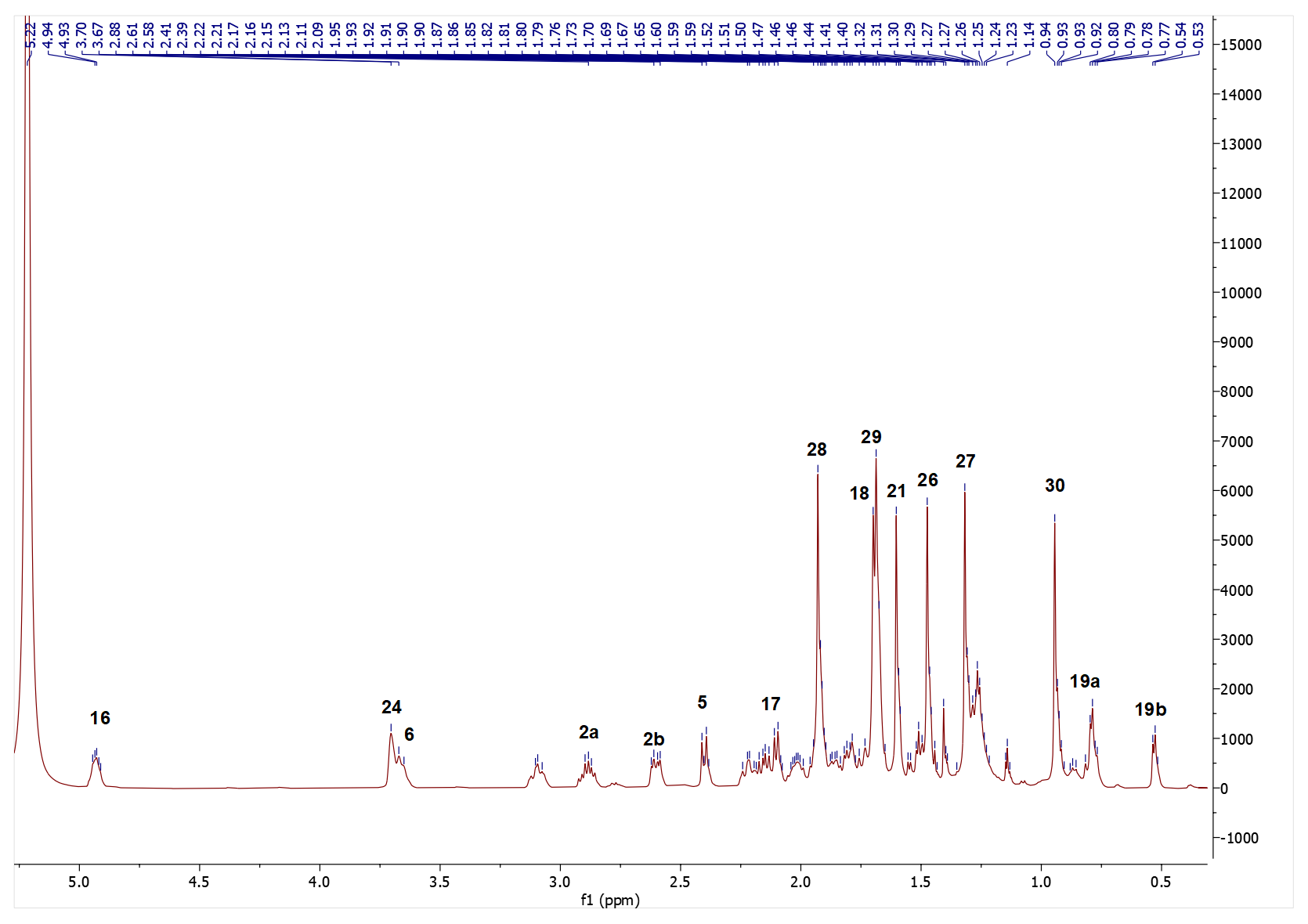


Fig. S 10. ^1^H-NMR spectrum of compound **5**.


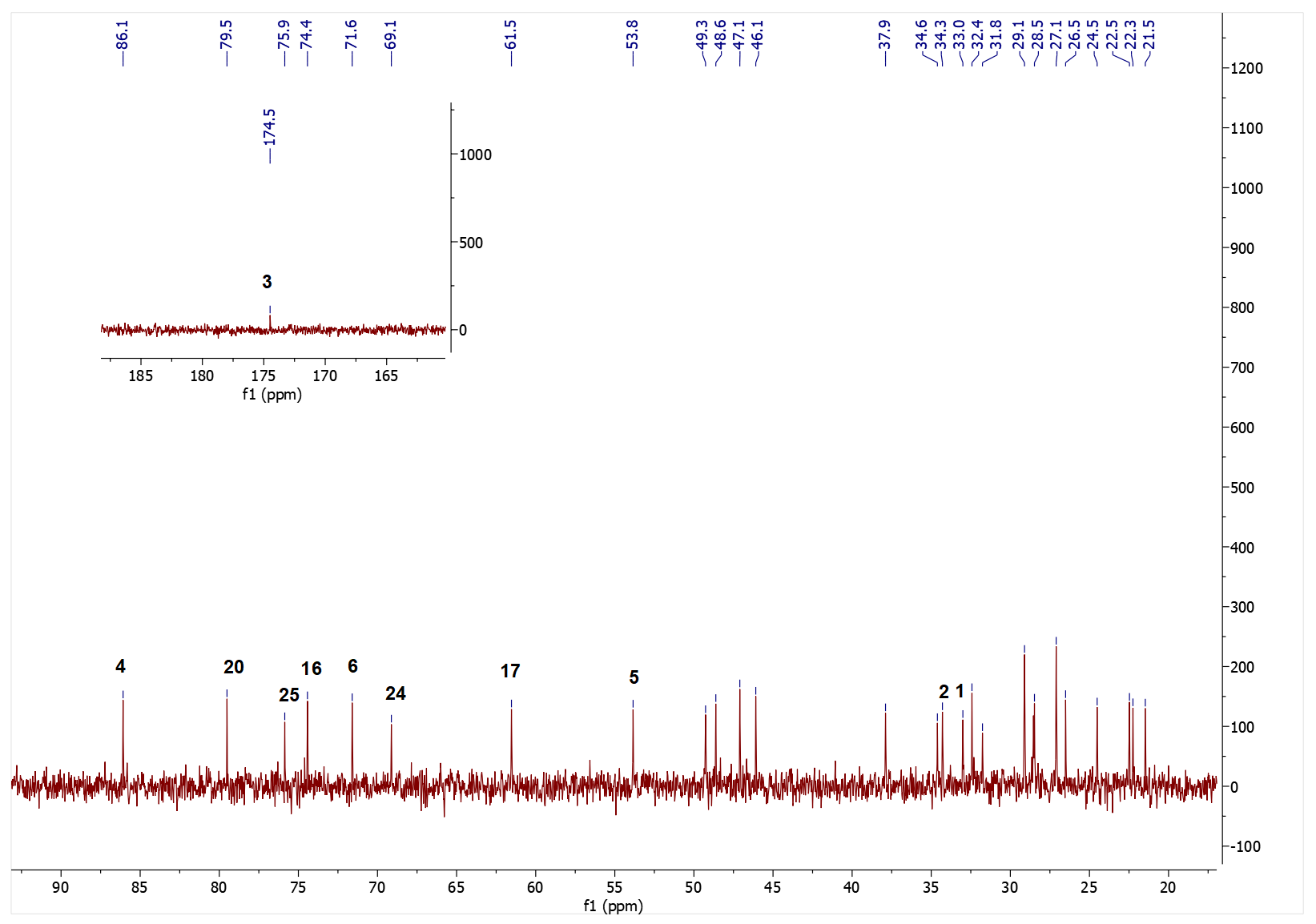


Fig. S 11. ^13^C-NMR spectrum of compound **5**.


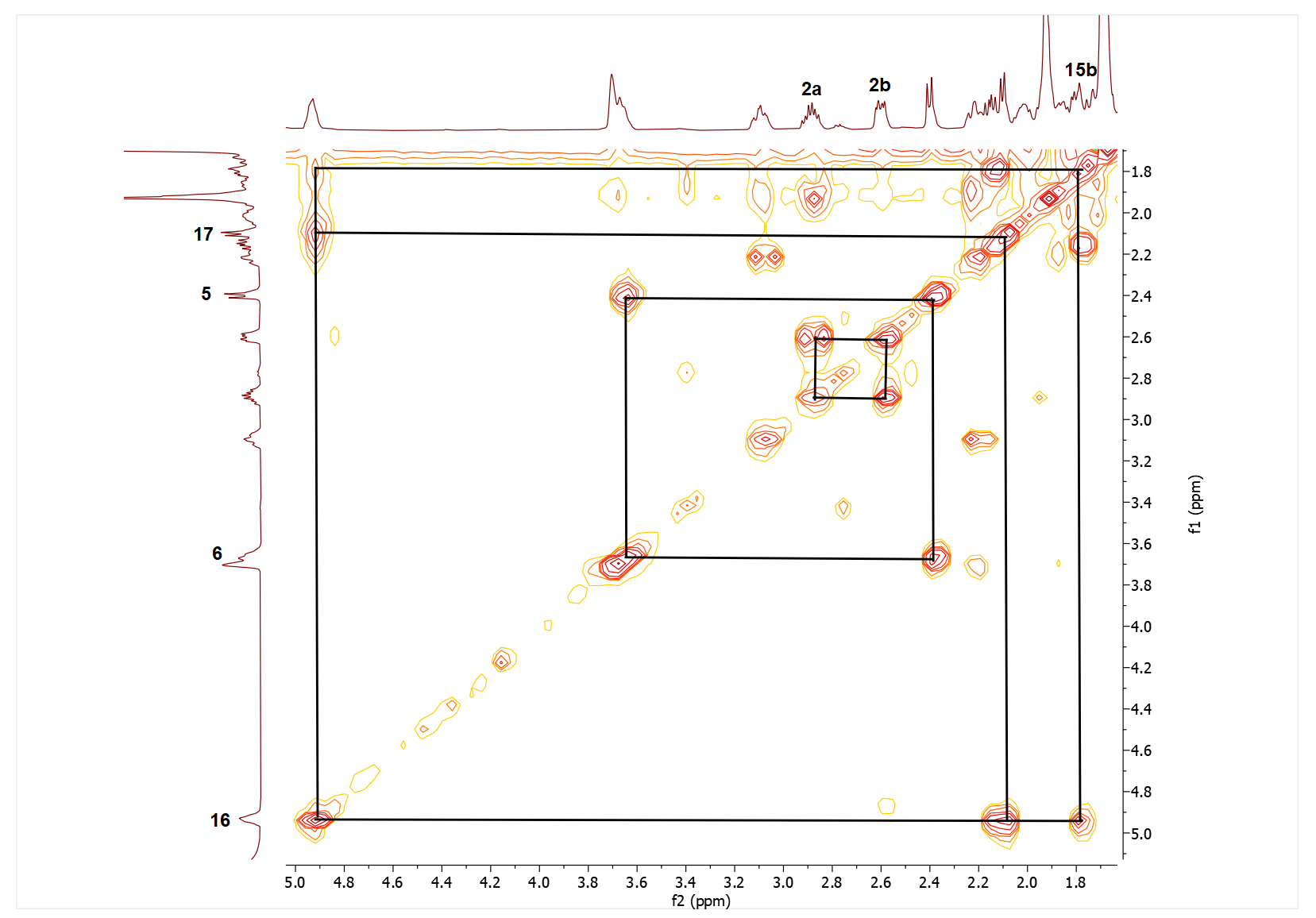


Fig. S 12. ^1^H-^1^H COSY spectrum of compound **5**.


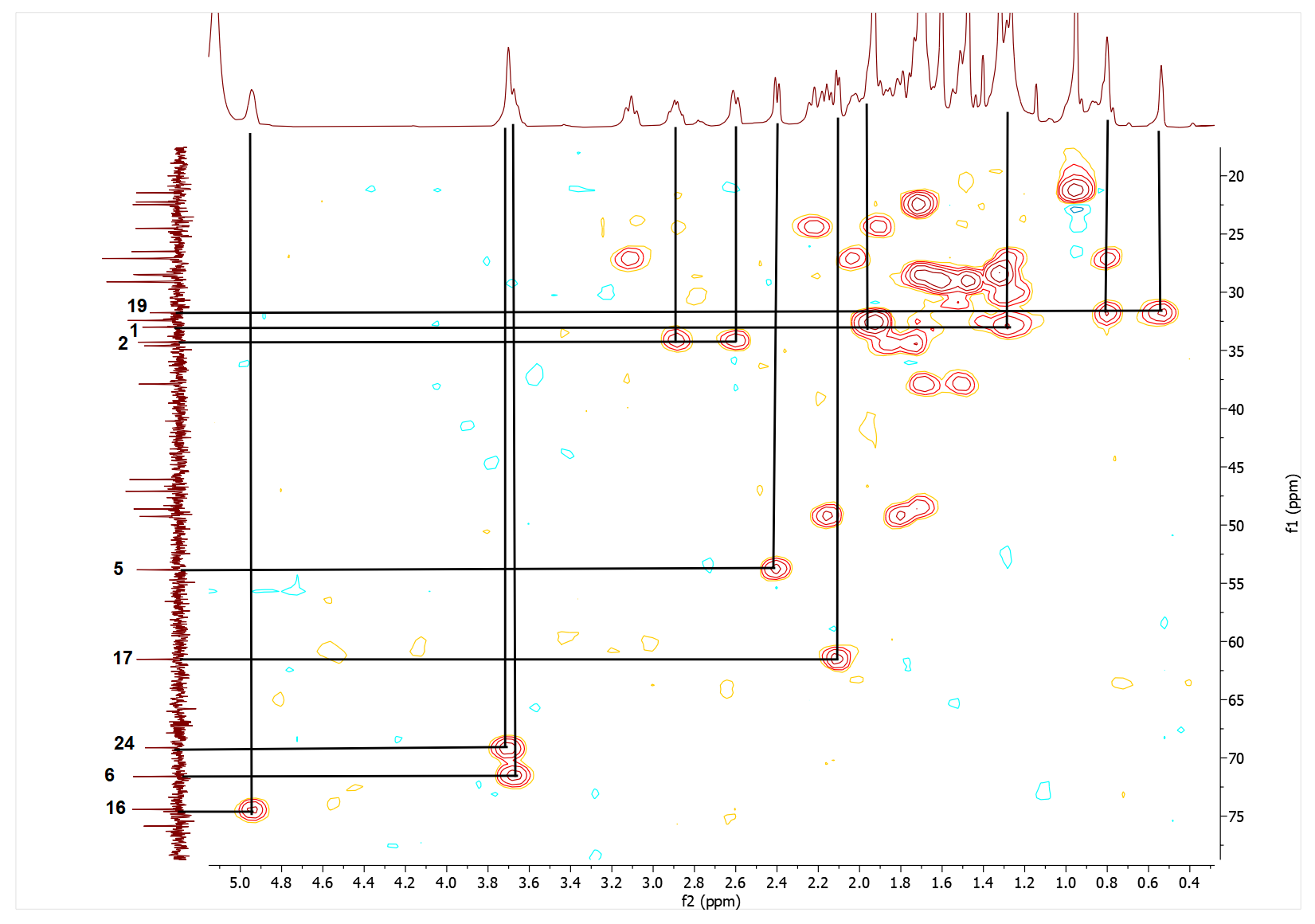


Fig. S 13. HSQC spectrum of compound **5**.


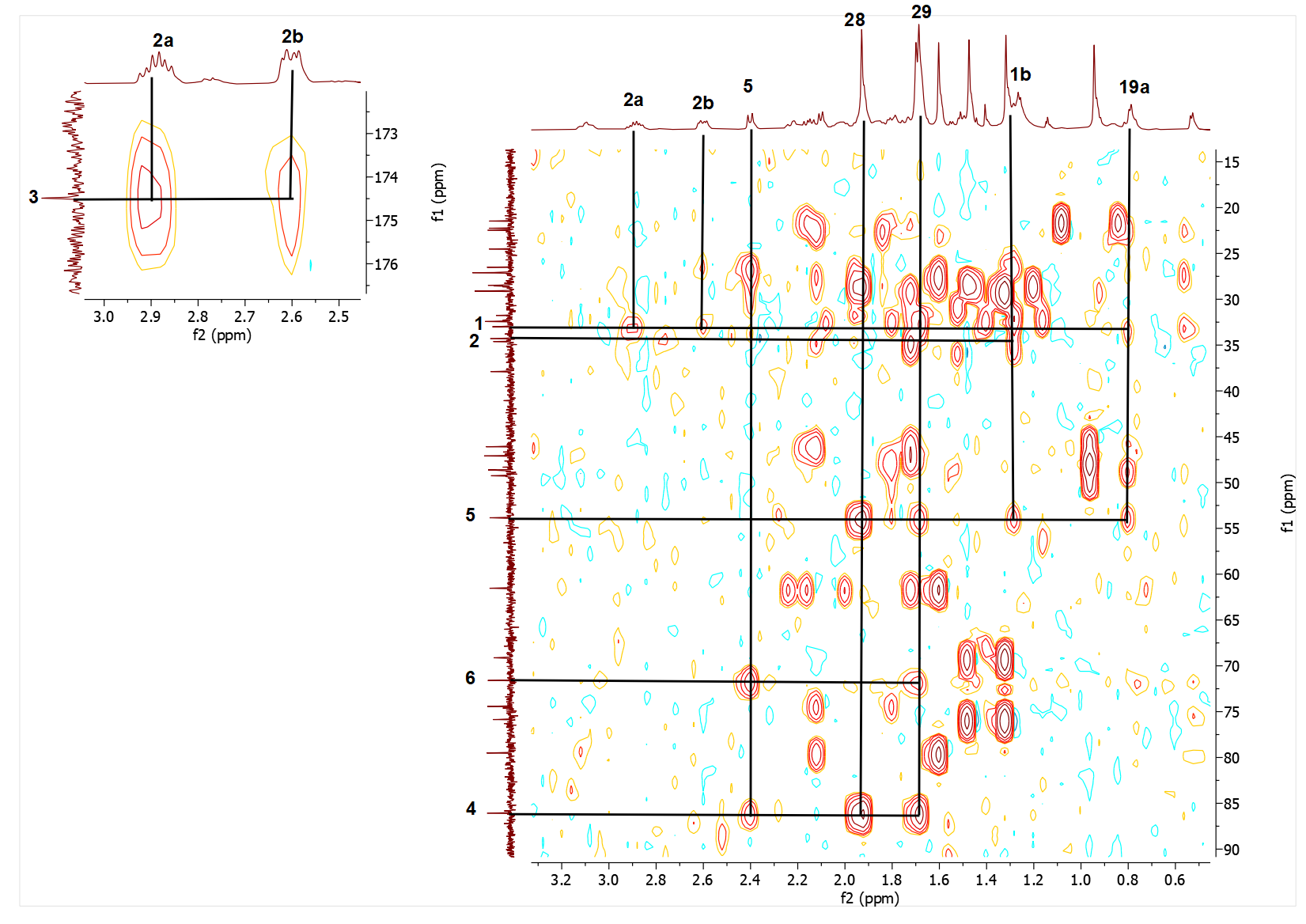

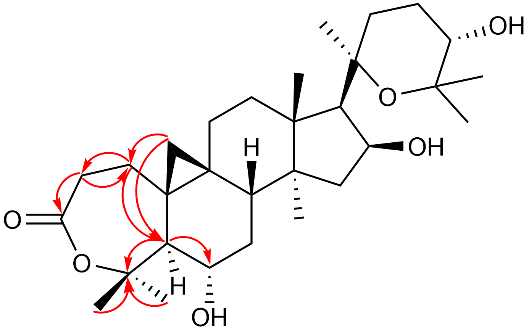


Fig. S 14. HMBC spectrum of compound **5**.

Fig. S 15. Chemical structure of compound **6**.


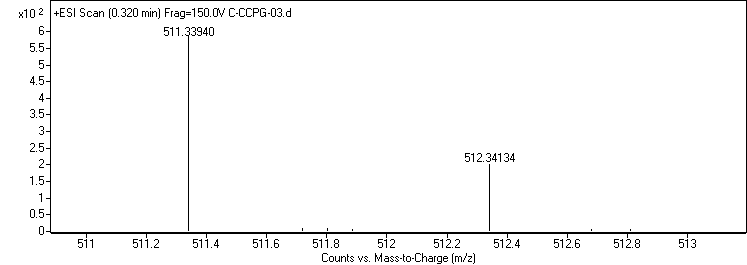


**[M+Na]^+^**

Fig. S 16. HR-ESI-MS spectrum of compound **6**.


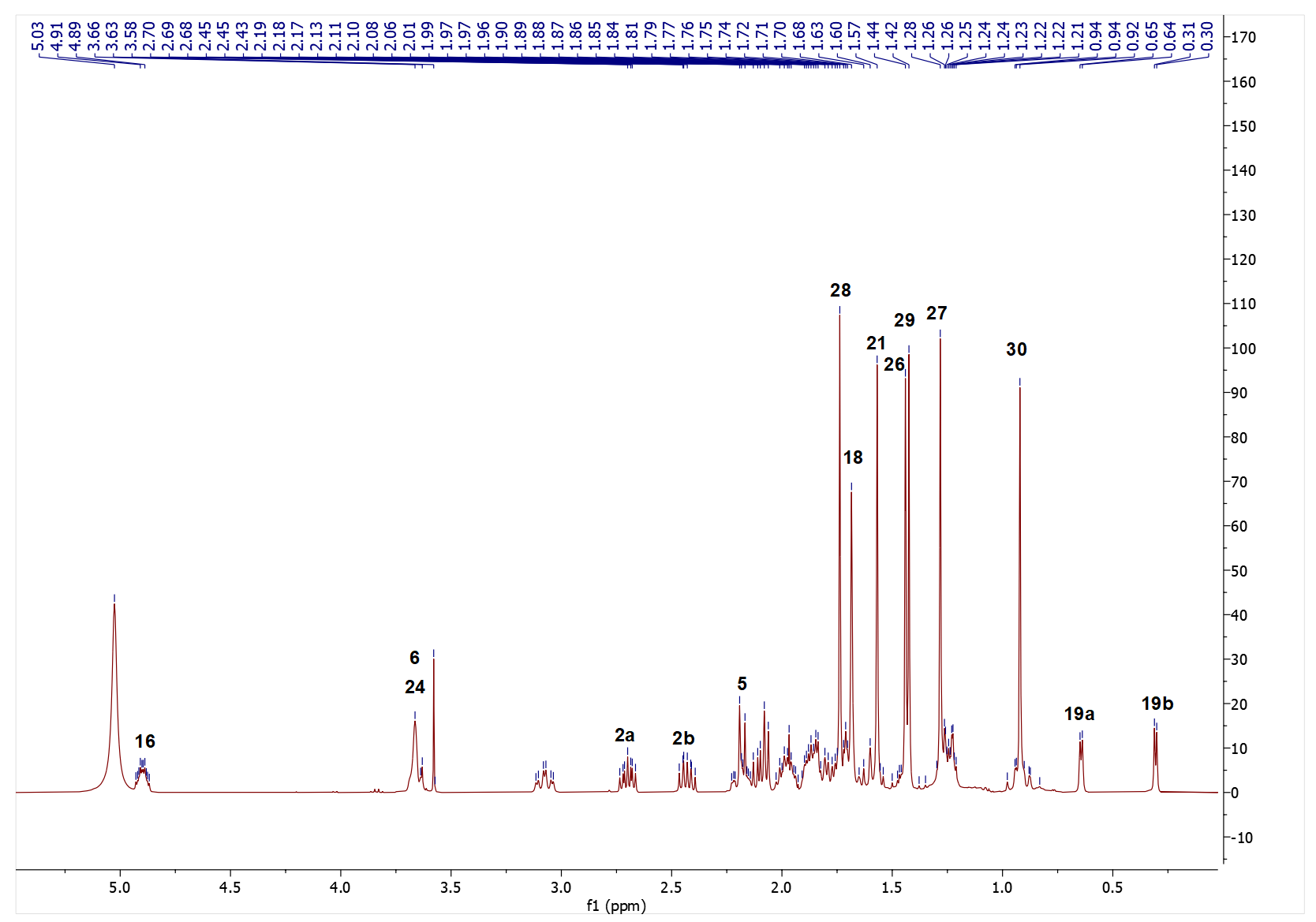


Fig. S 17. ^1^H-NMR spectrum of compound **6**.


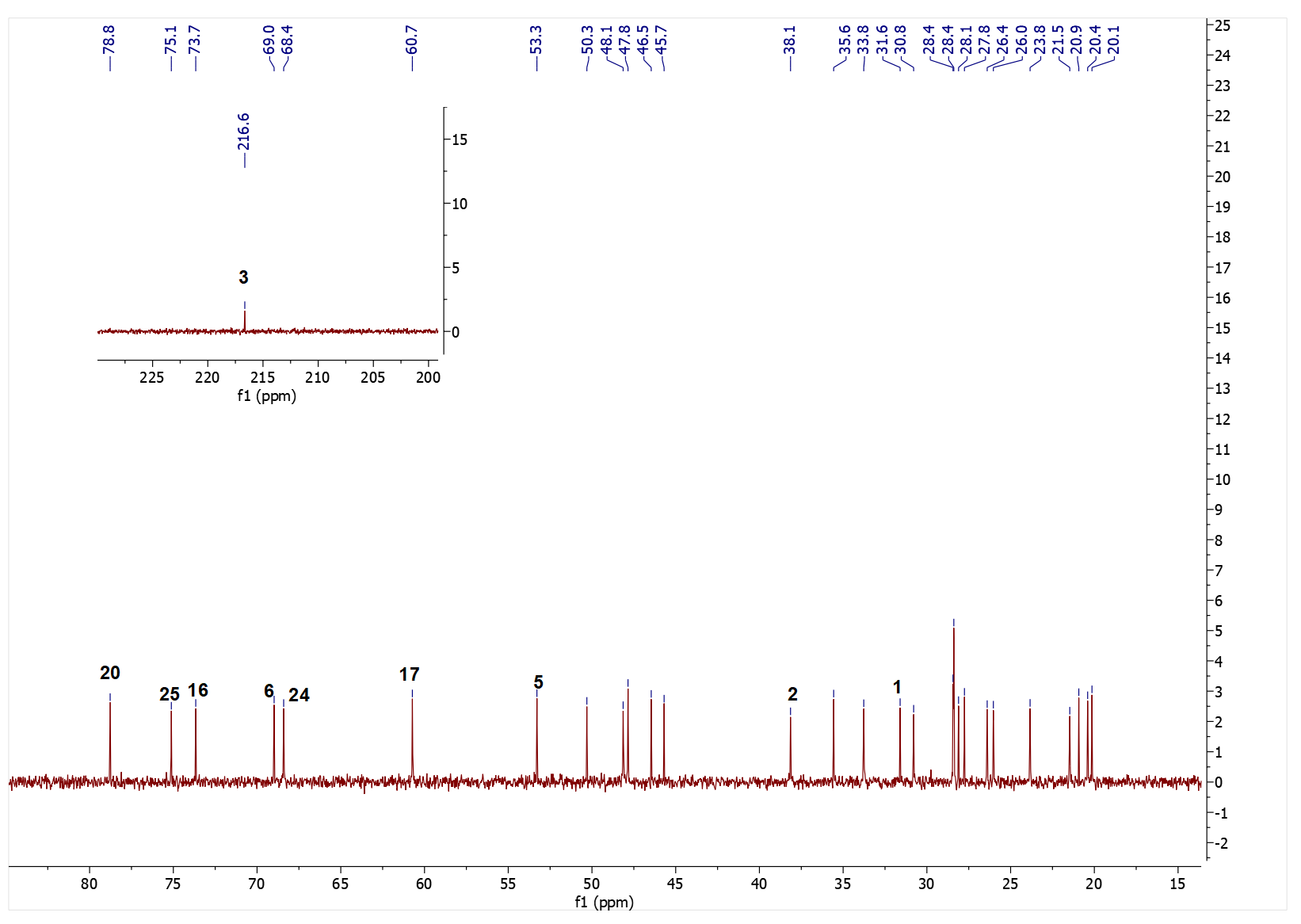


Fig. S 18. ^13^C-NMR spectrum of compound **6**.


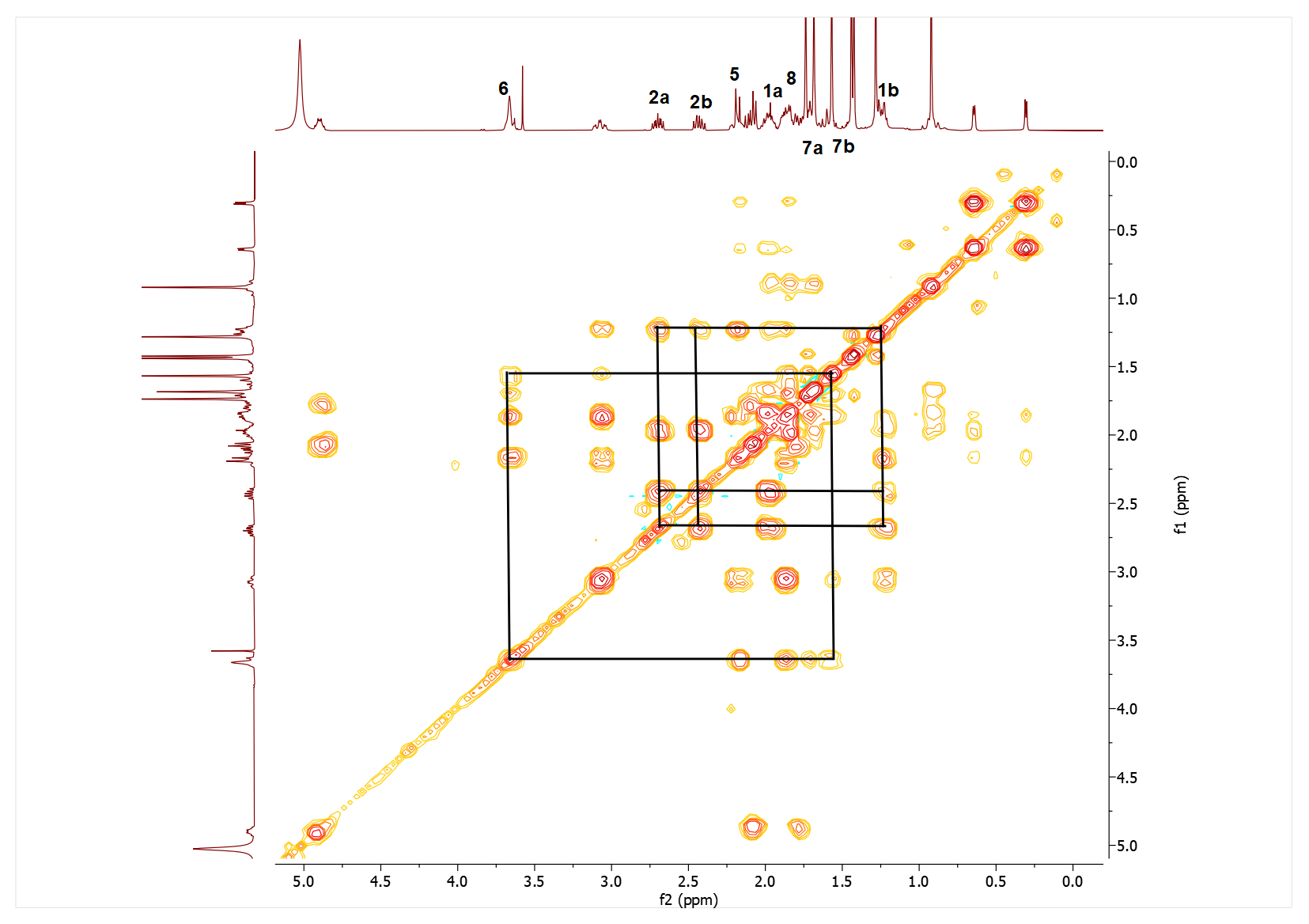


Fig. S 19. ^1^H-^1^H COSY spectrum of compound **6**.


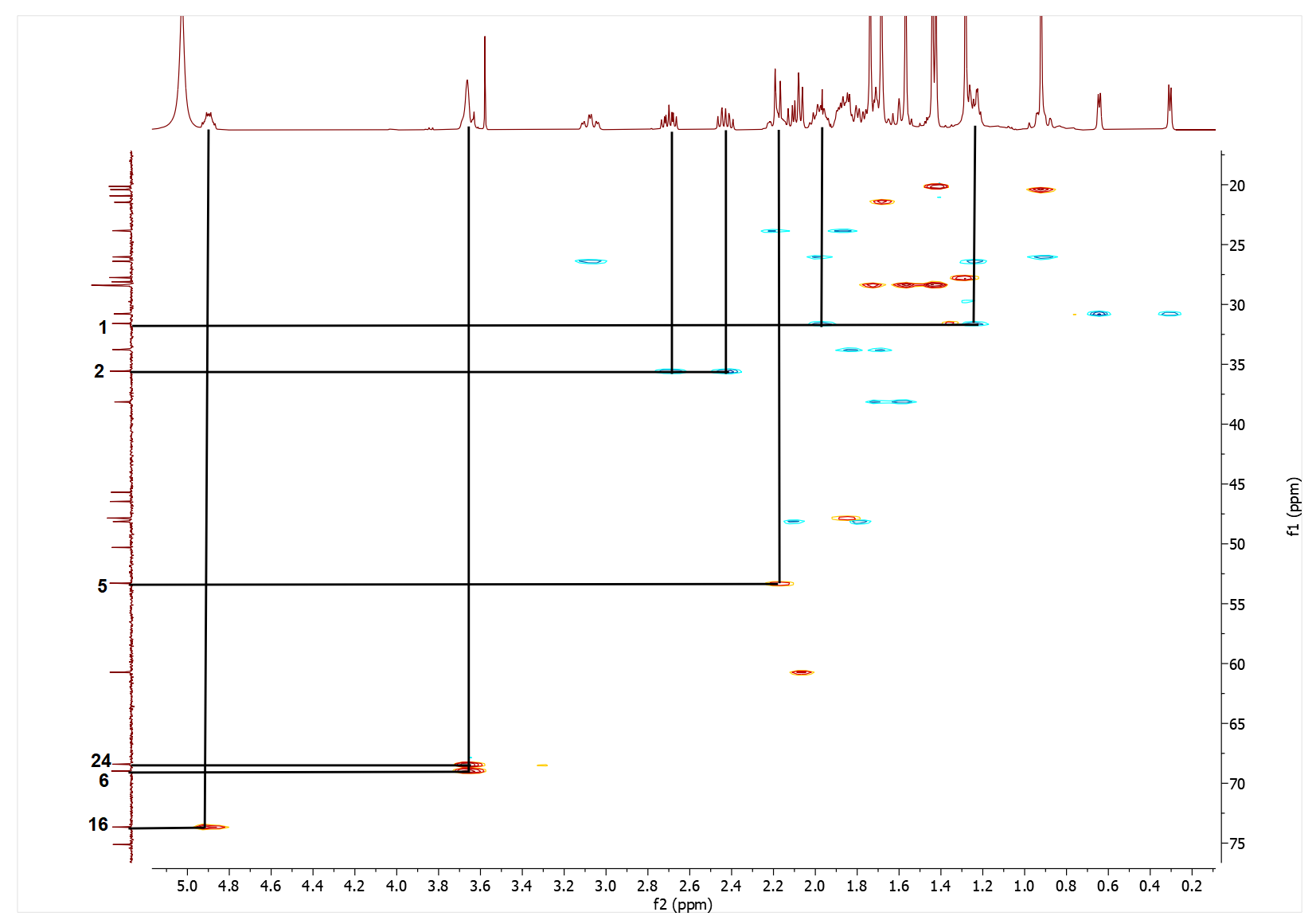


Fig. S 20. HSQC spectrum of compound **6**.


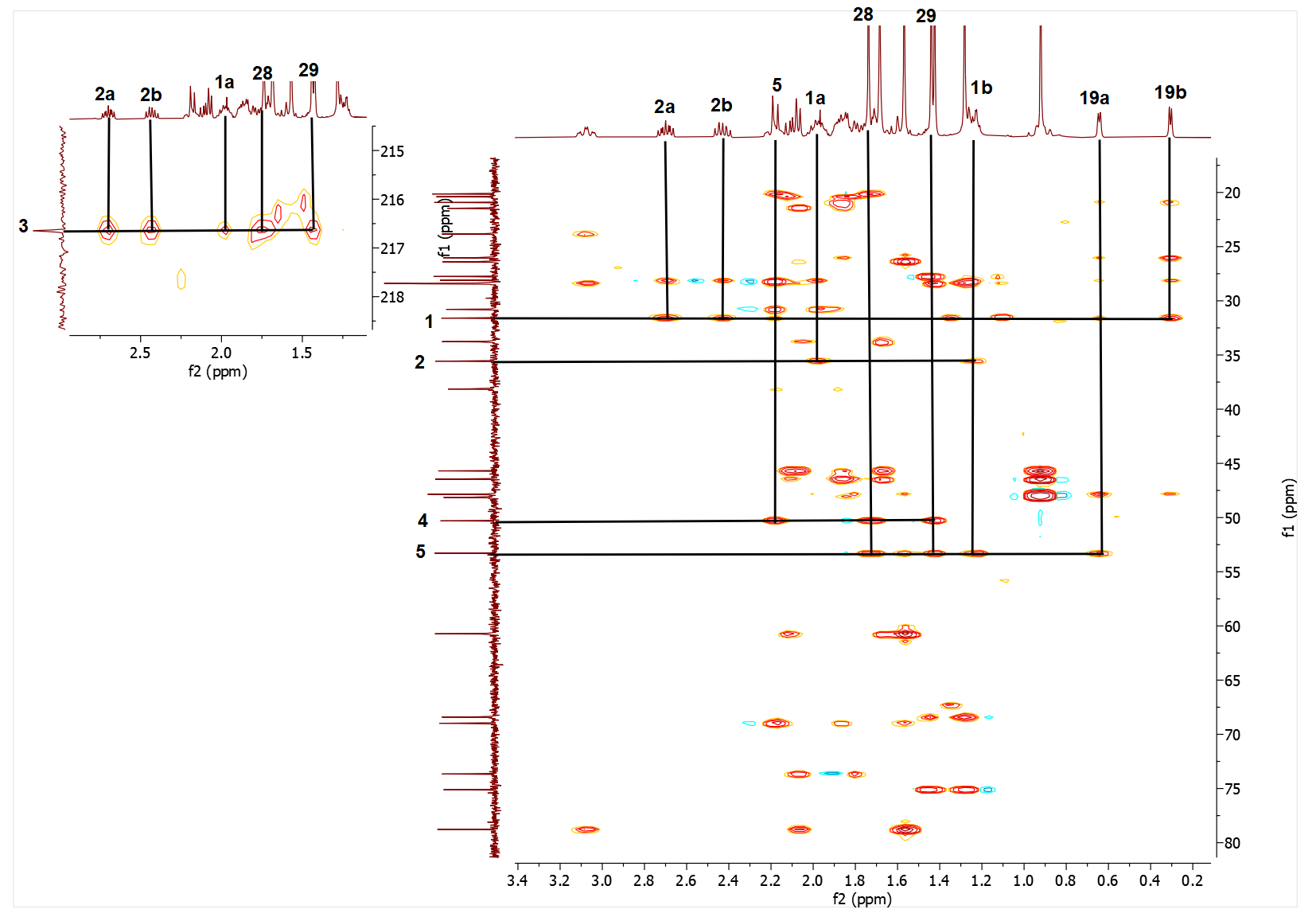

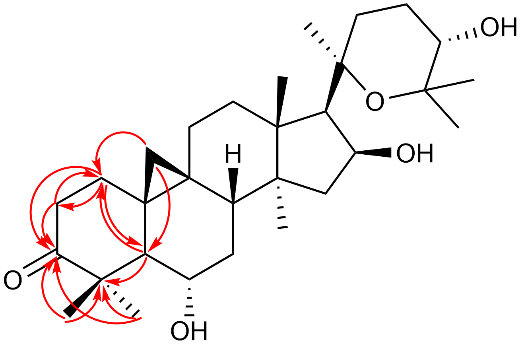


Fig. S 21. HMBC spectrum of compound **6**.

Fig. S 22. Chemical structure of compound **7**.


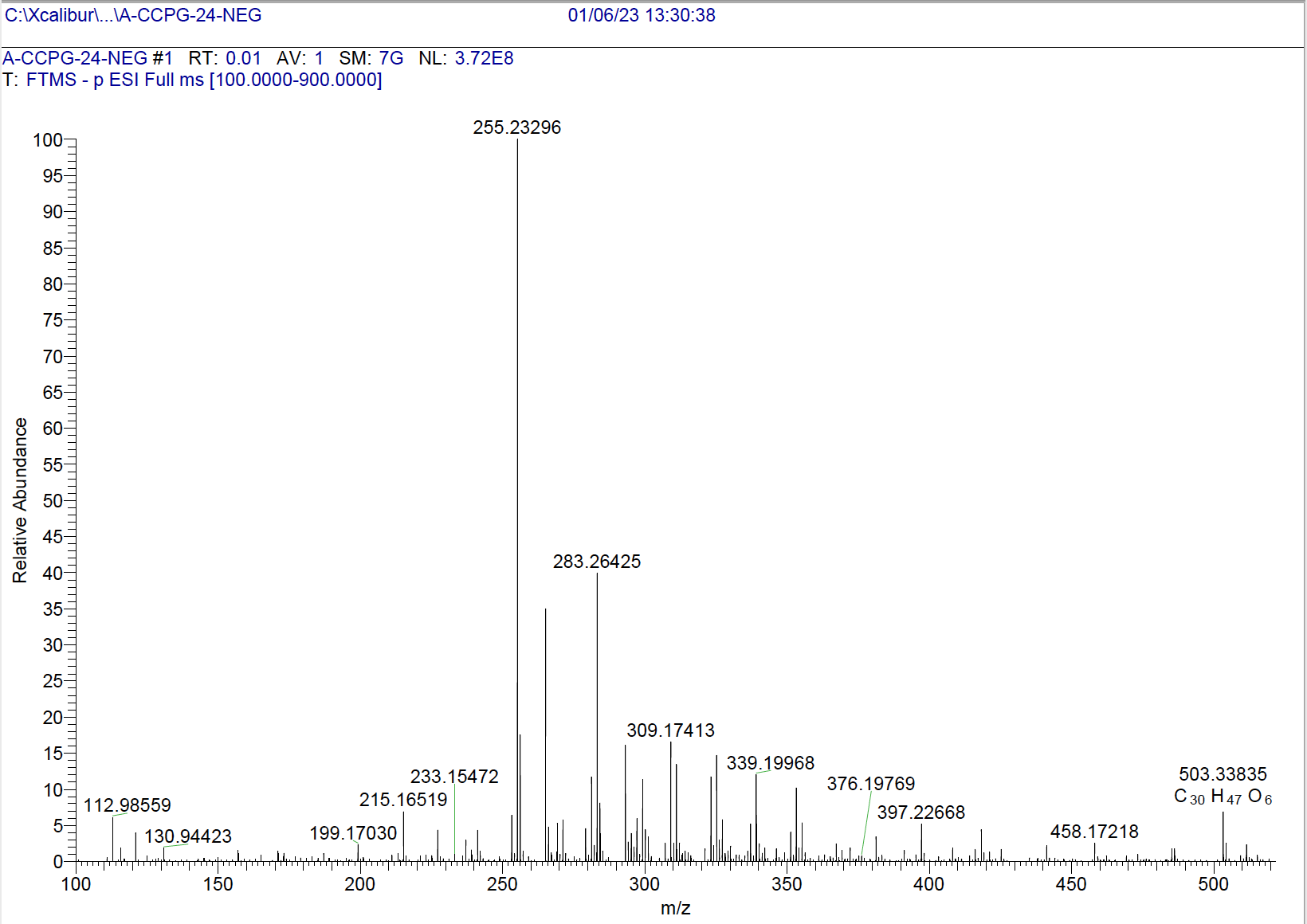


**[M-H]^-^**

Fig. S 23. HR-ESI-MS spectrum of compound **7**.


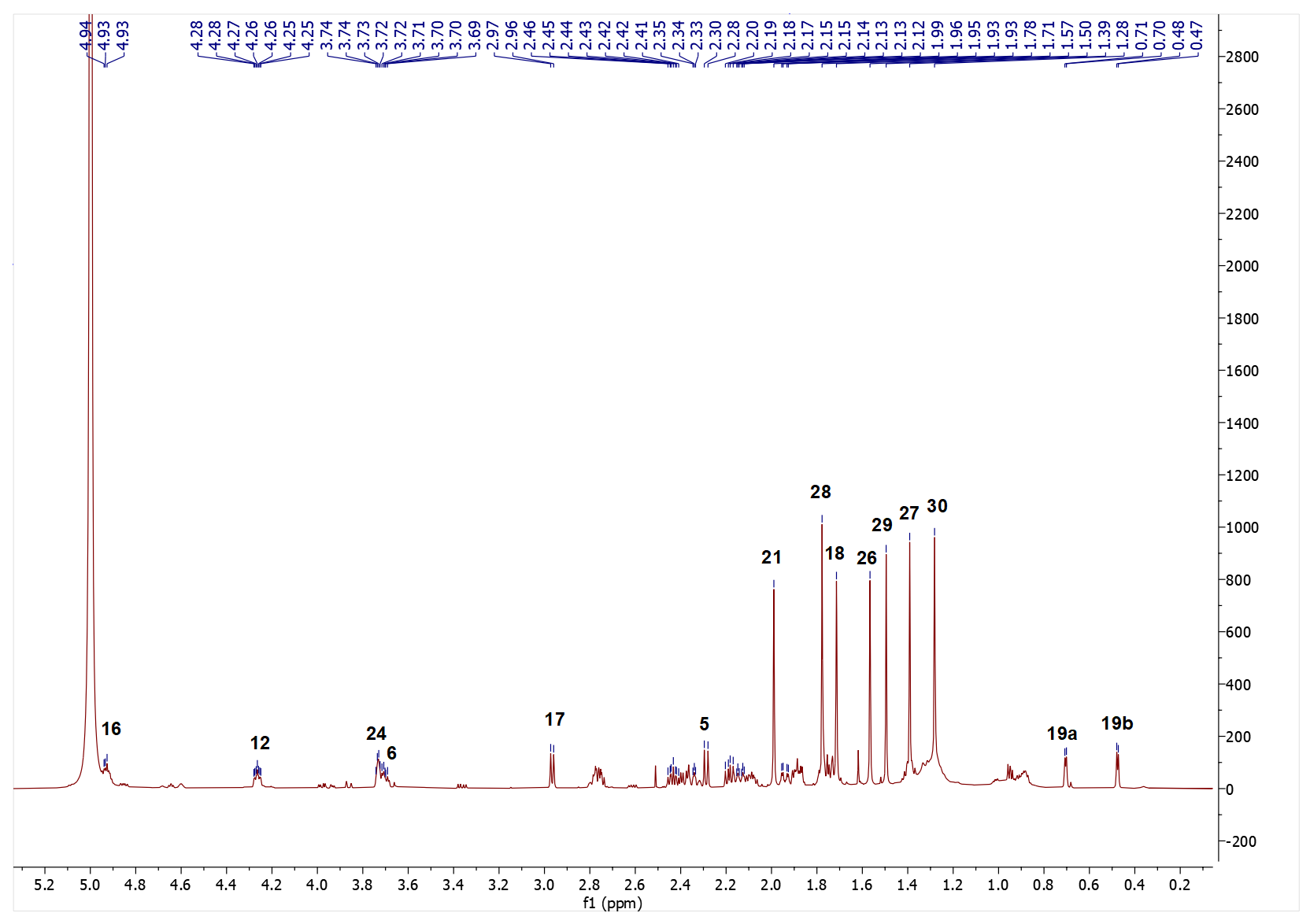


Fig. S 24. ^1^H-NMR spectrum of compound **7**.


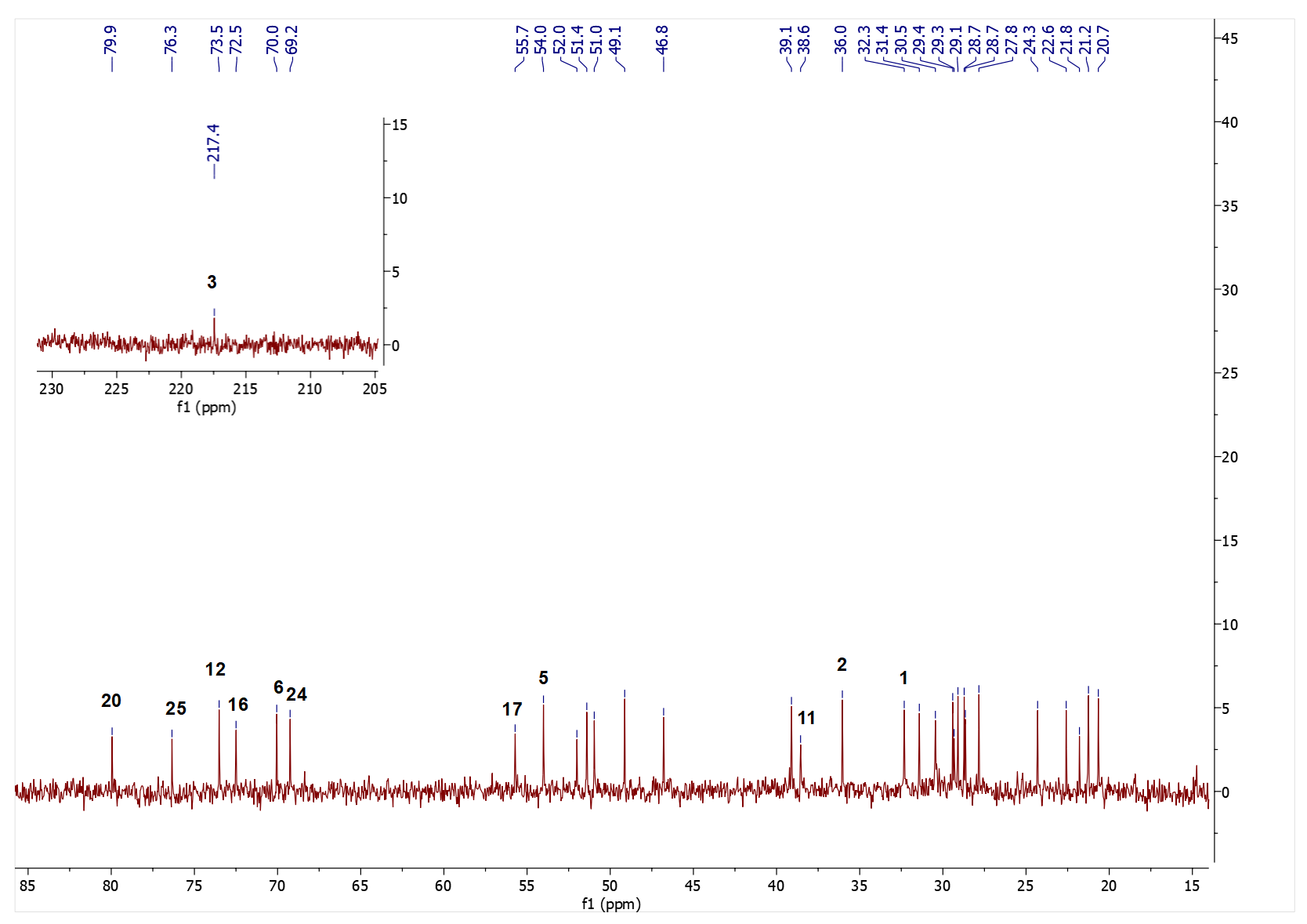


Fig. S 25. ^13^C-NMR spectrum of compound **7**.


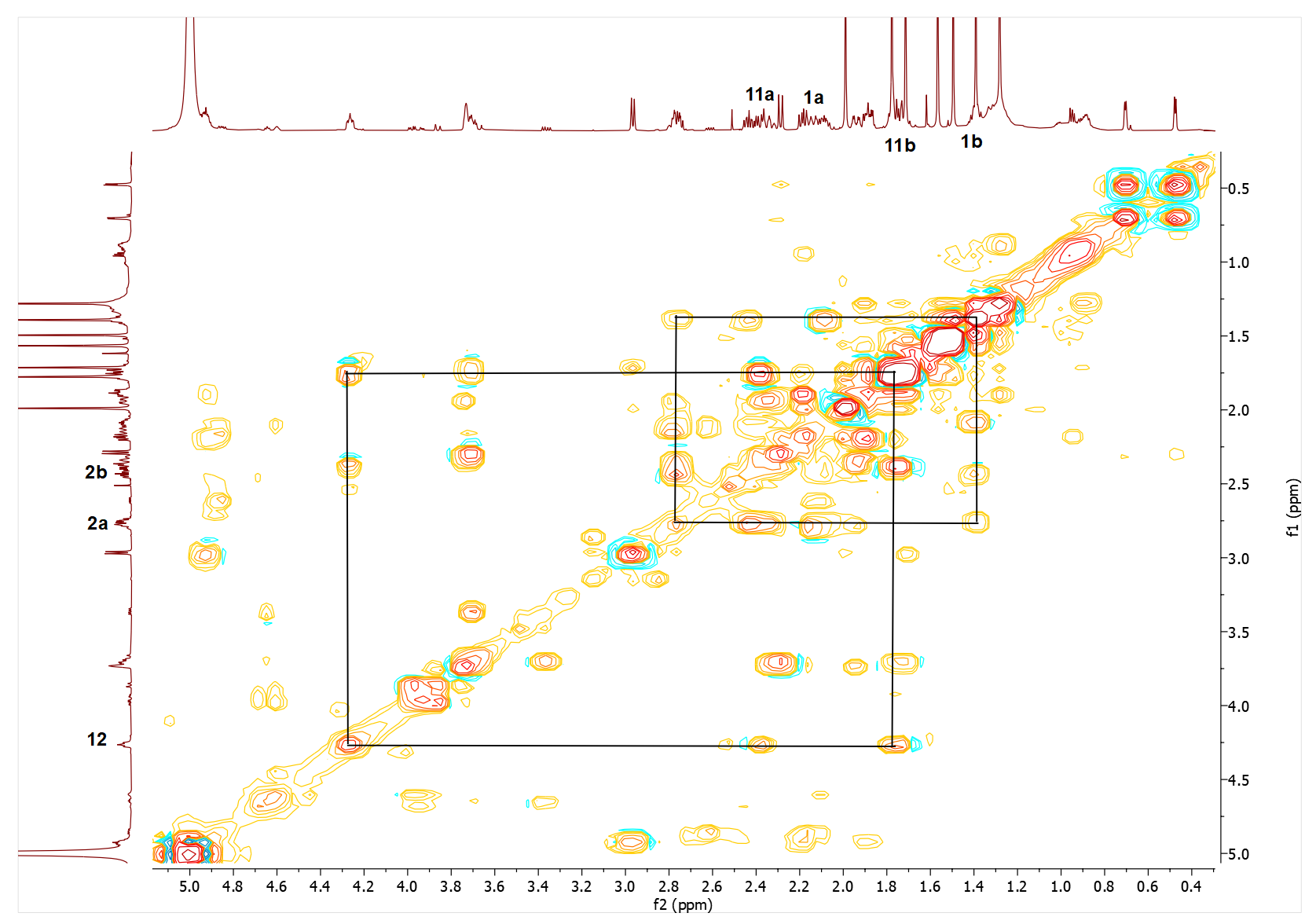


Fig. S 26. ^1^H-^1^H COSY spectrum of compound **7**.


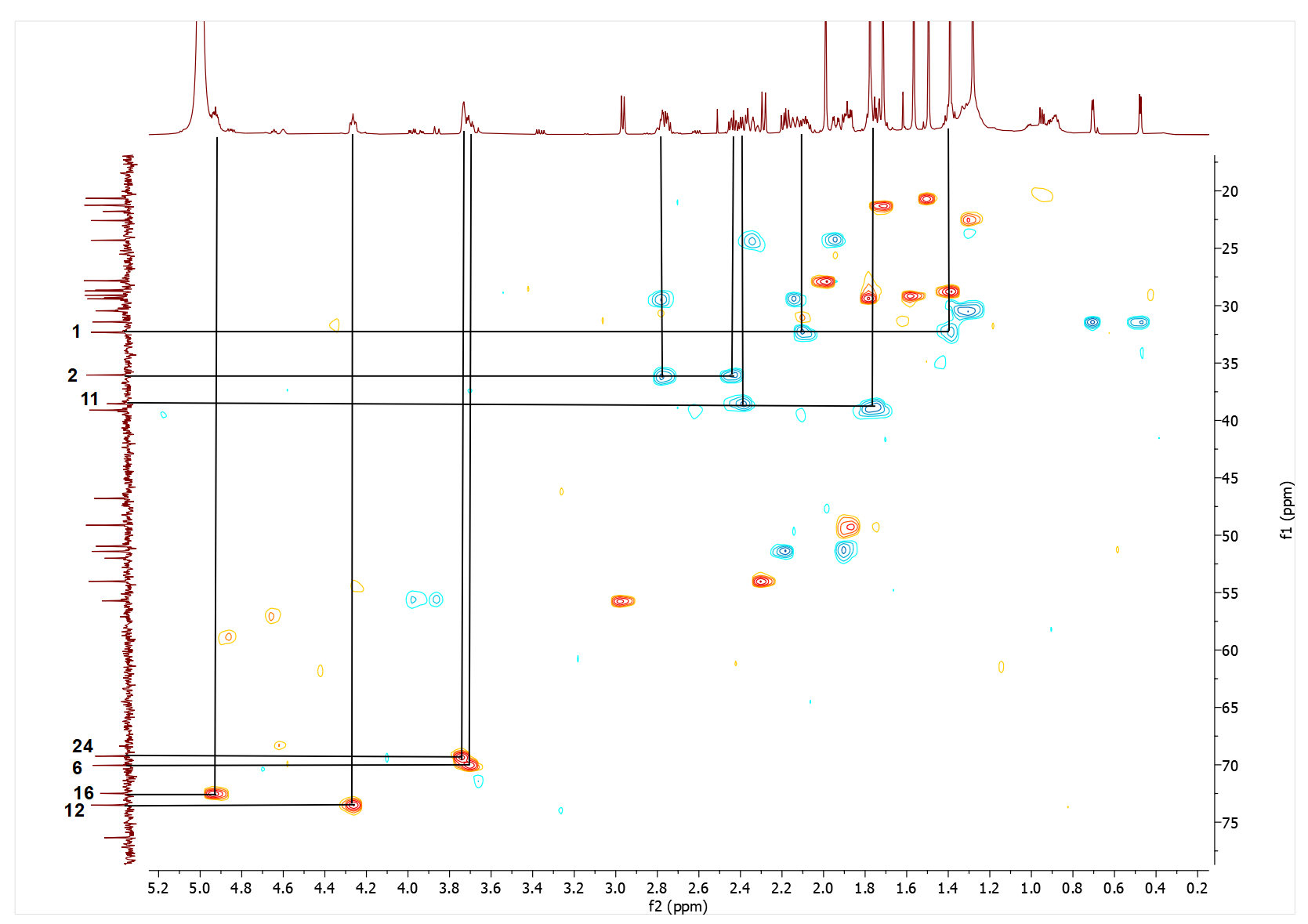


Fig. S 27. HSQC spectrum of compound **7**.


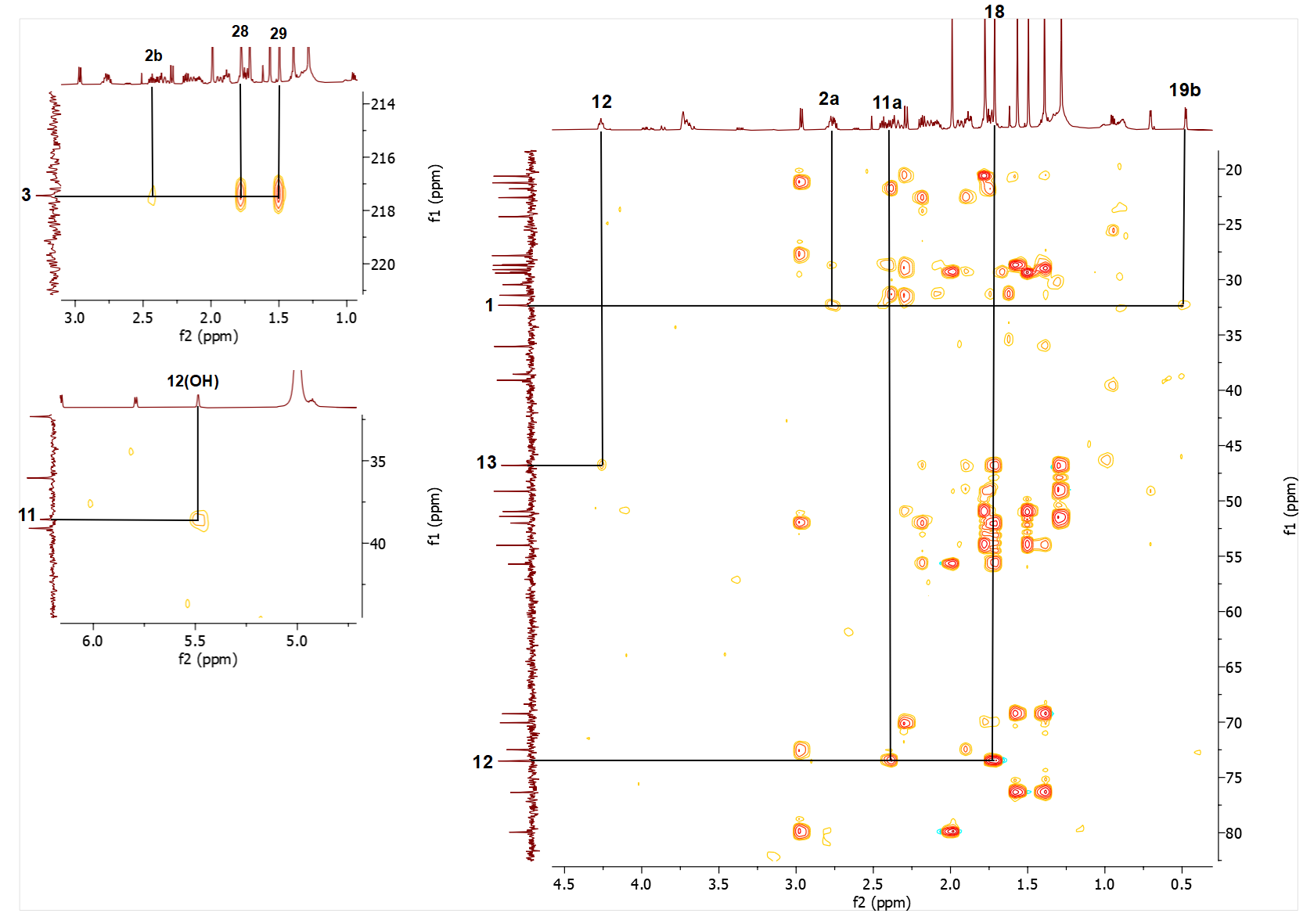

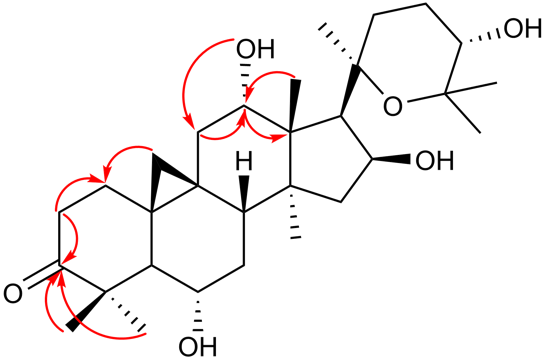


Fig. S 28. HMBC spectrum of compound **7**.

Fig. S 29. Chemical structure of compound **8**.


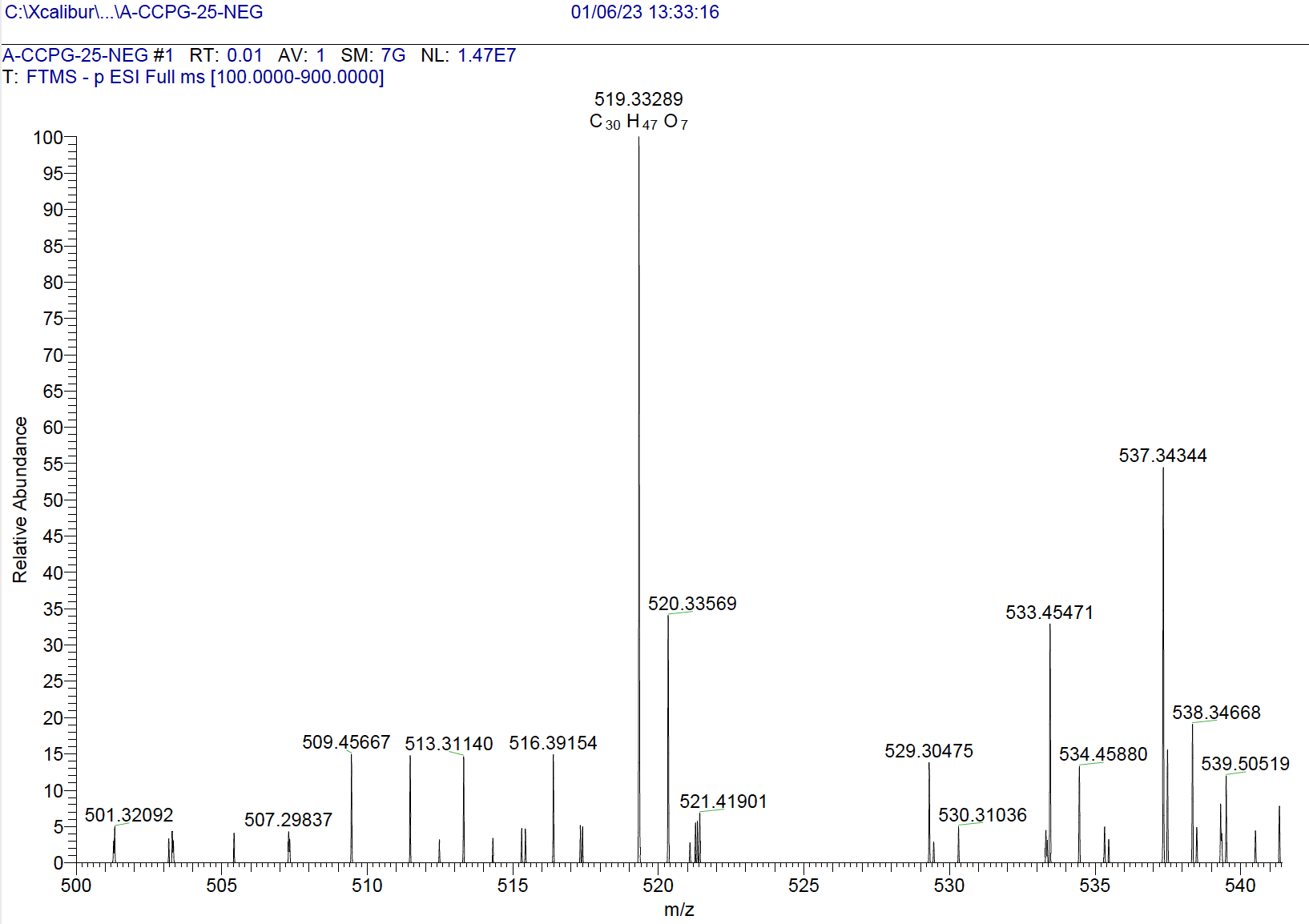


**[M-H]^-^**

Fig. S 30. HR-ESI-MS spectrum of compound **8**.


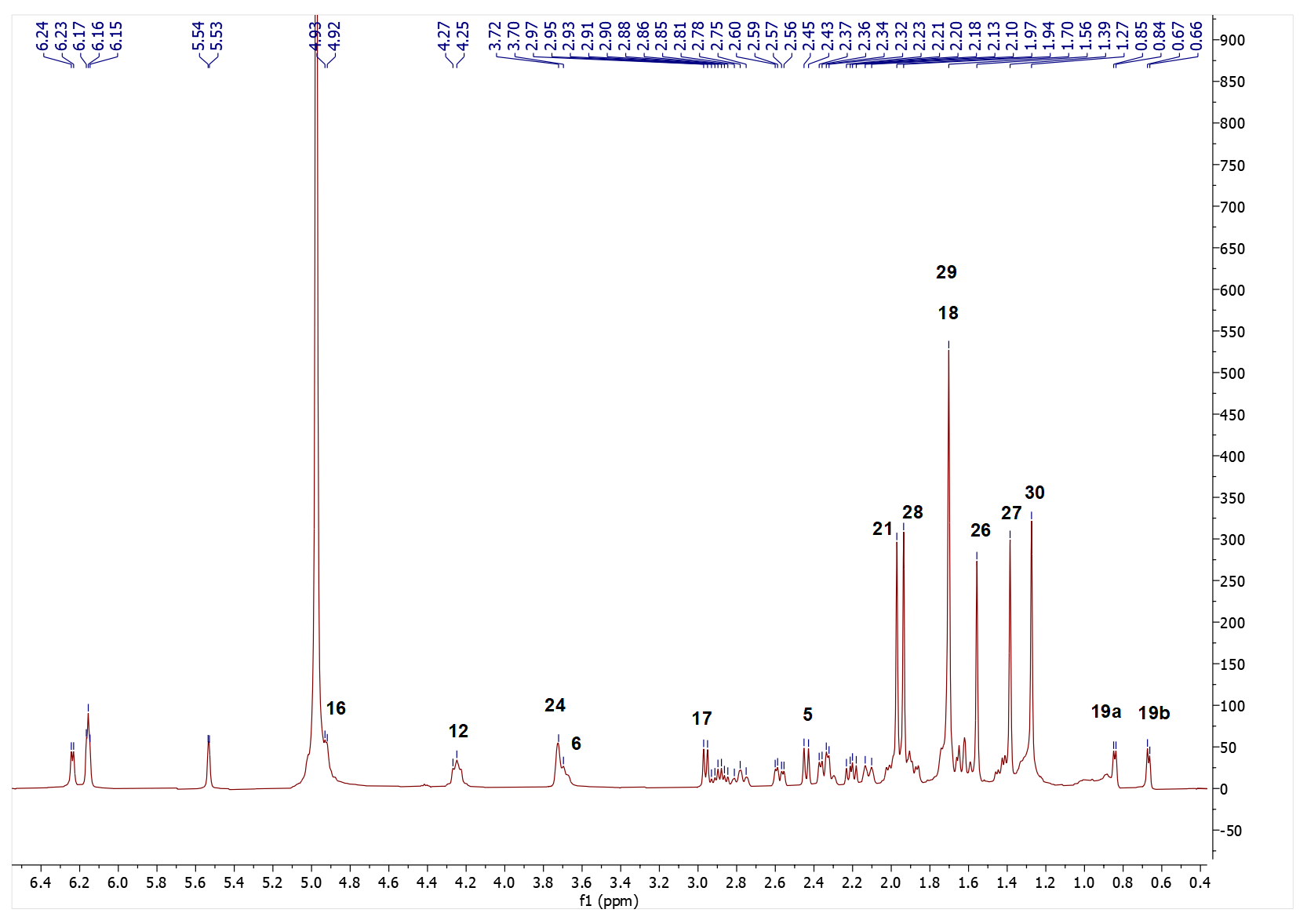


Fig. S 31. ^1^H-NMR spectrum of compound **8**.


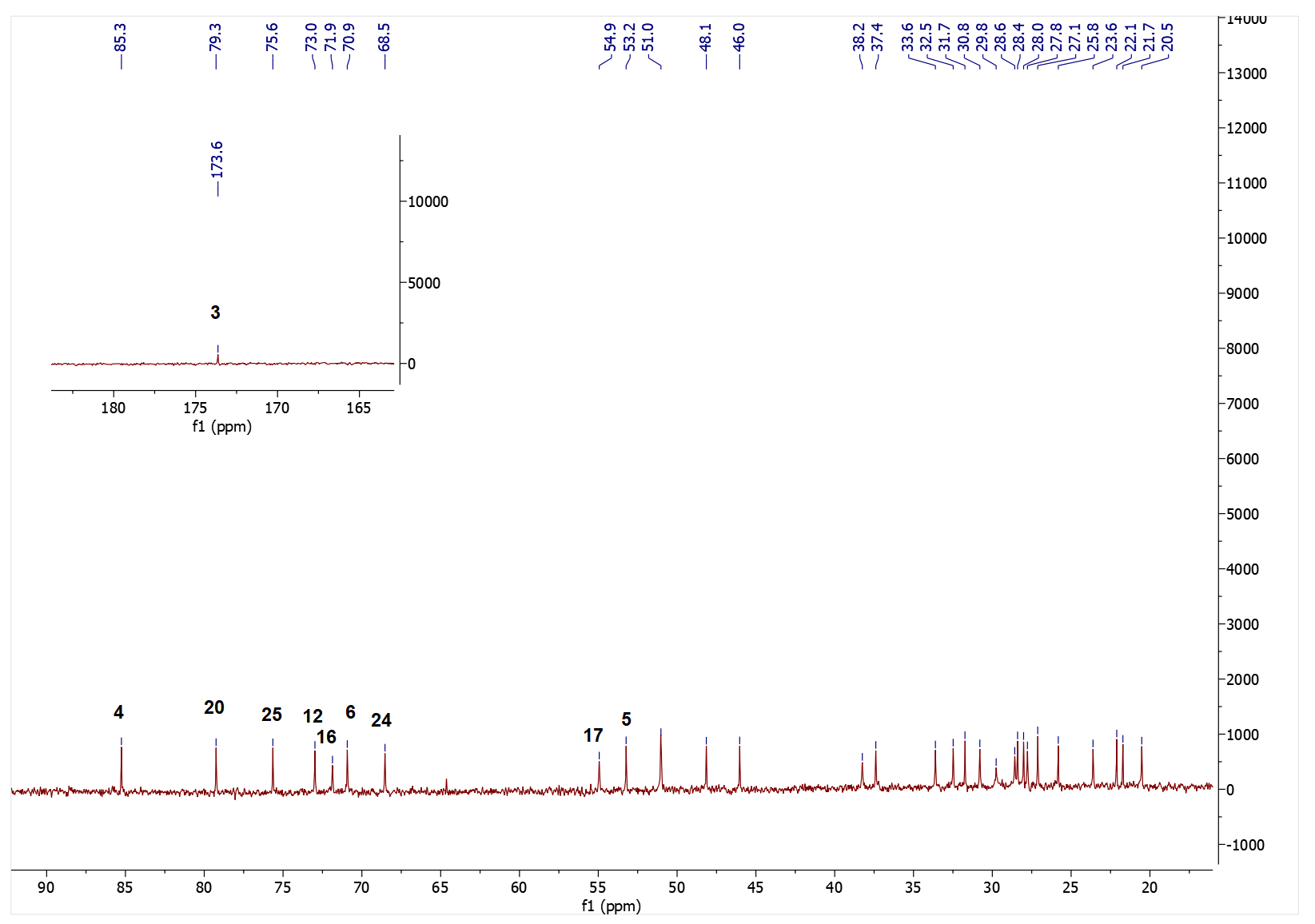


Fig. S 32. ^13^C-NMR spectrum of compound **8**.


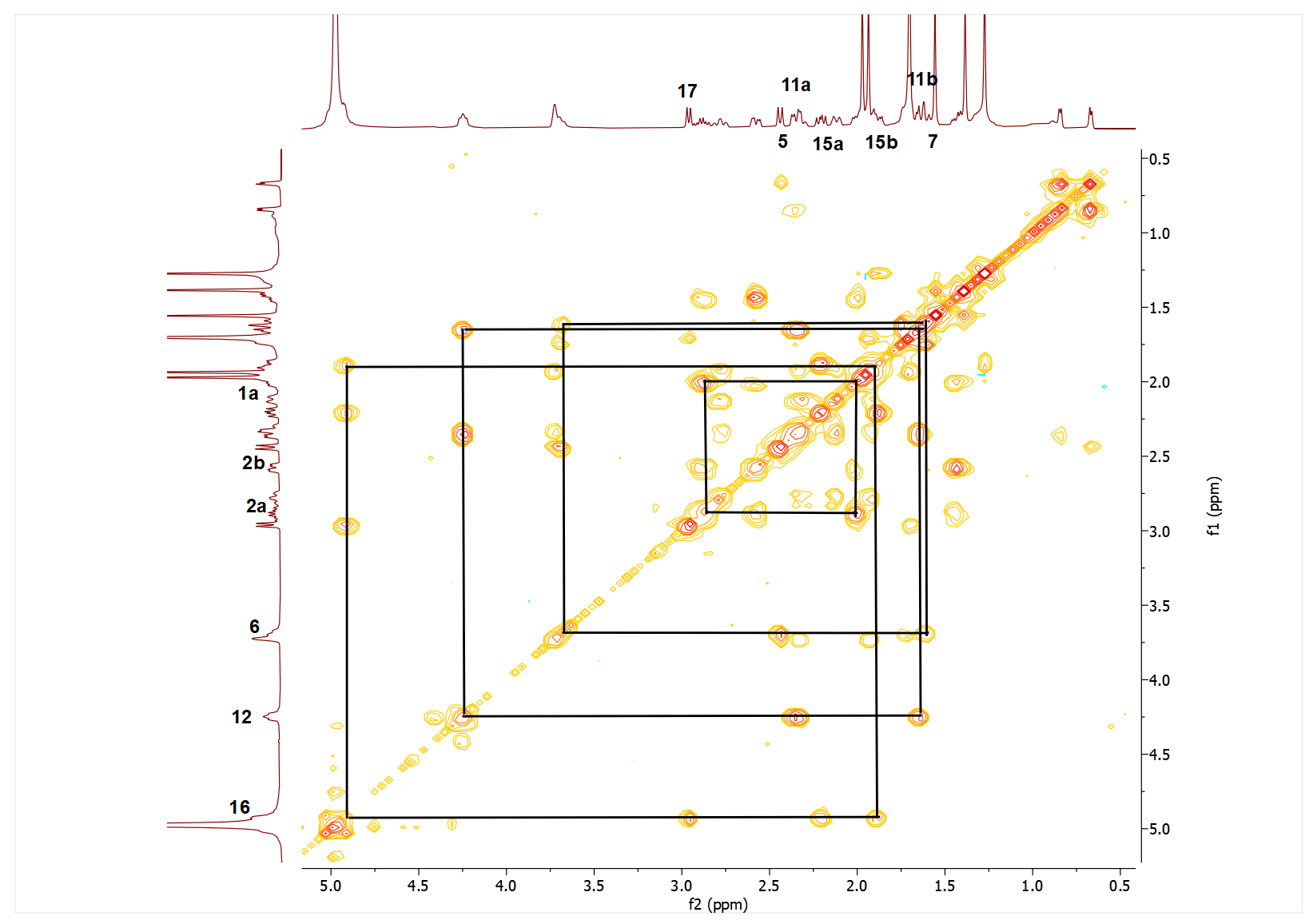


Fig. S 33. ^1^H-^1^H COSY spectrum of compound **8**.


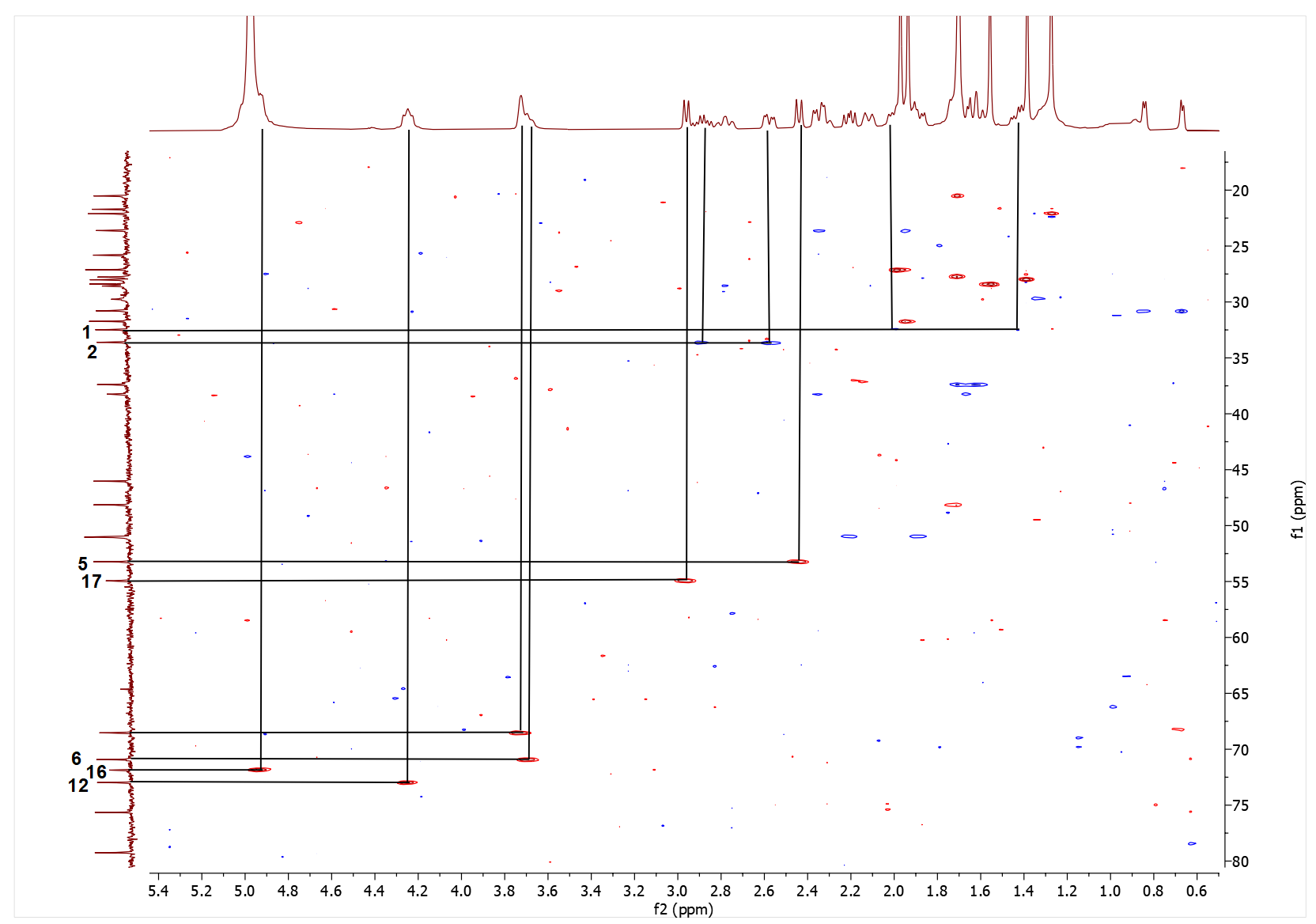


Fig. S 34. HSQC spectrum of compound **8**.


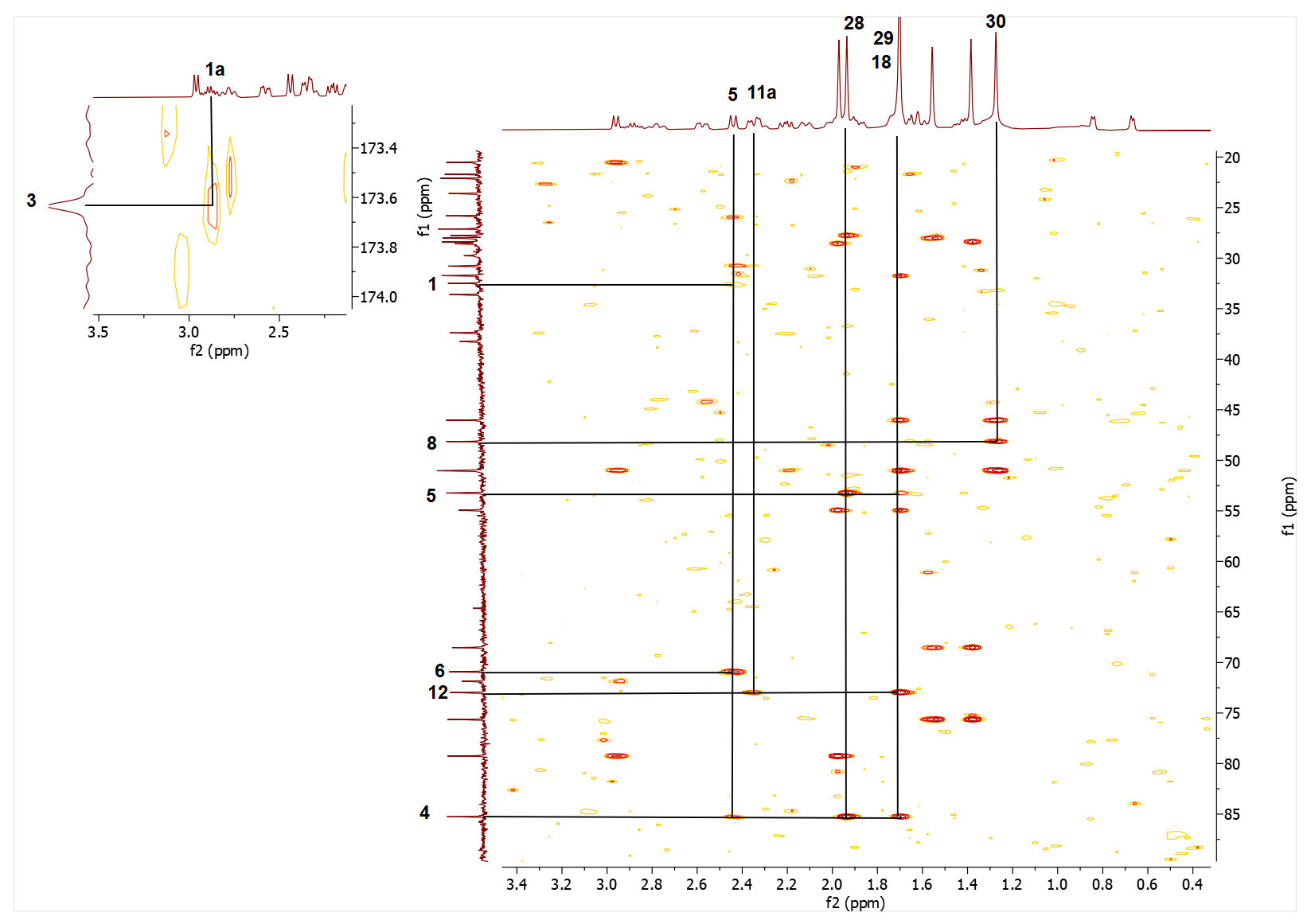


Fig. S 35. HMBC spectrum of compound **8**.


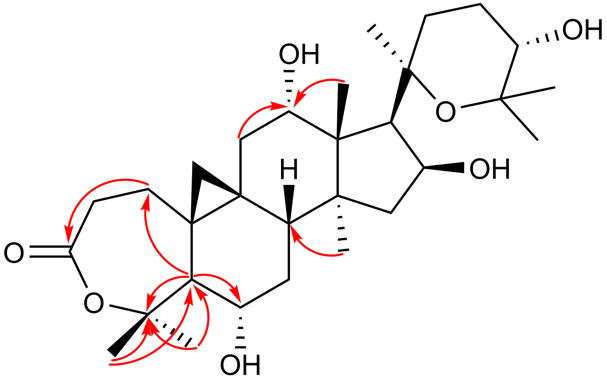

Fig. S 36. Chemical structure of compound **9**.


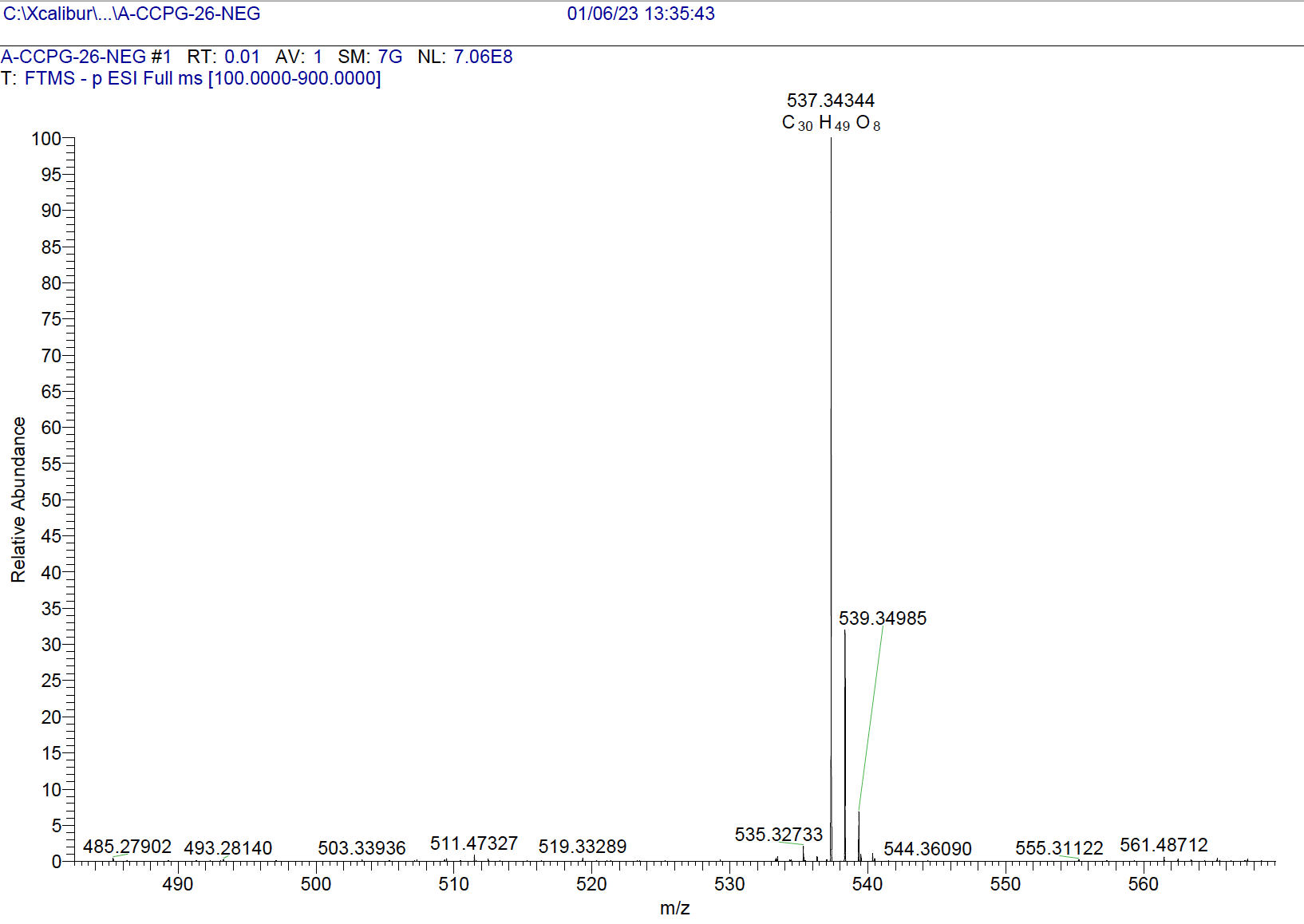


**[M-H]^-^**

Fig. S 37. HR-ESI-MS spectrum of compound **9**.


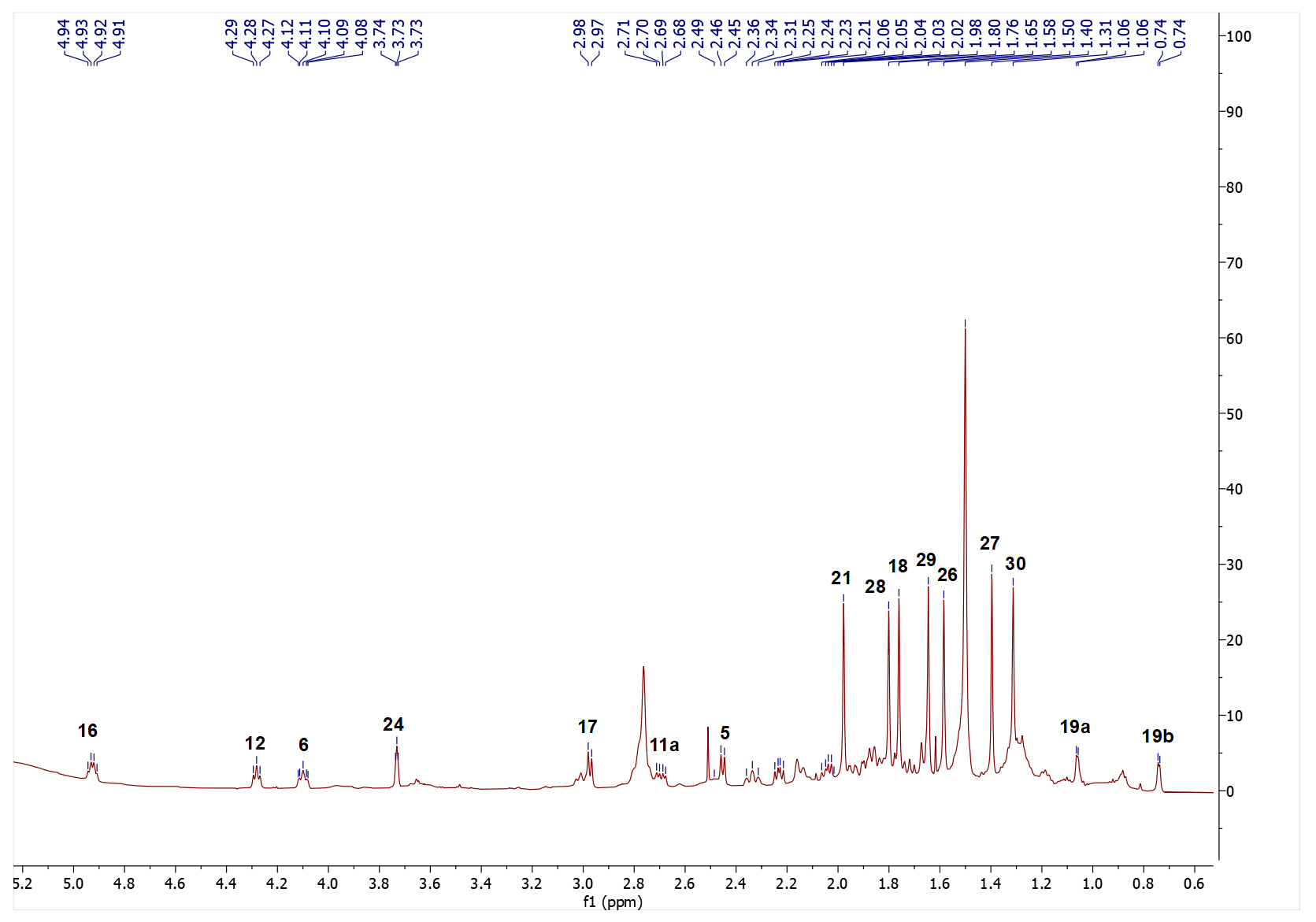


Fig. S 38. ^1^H-NMR spectrum of compound **9**.


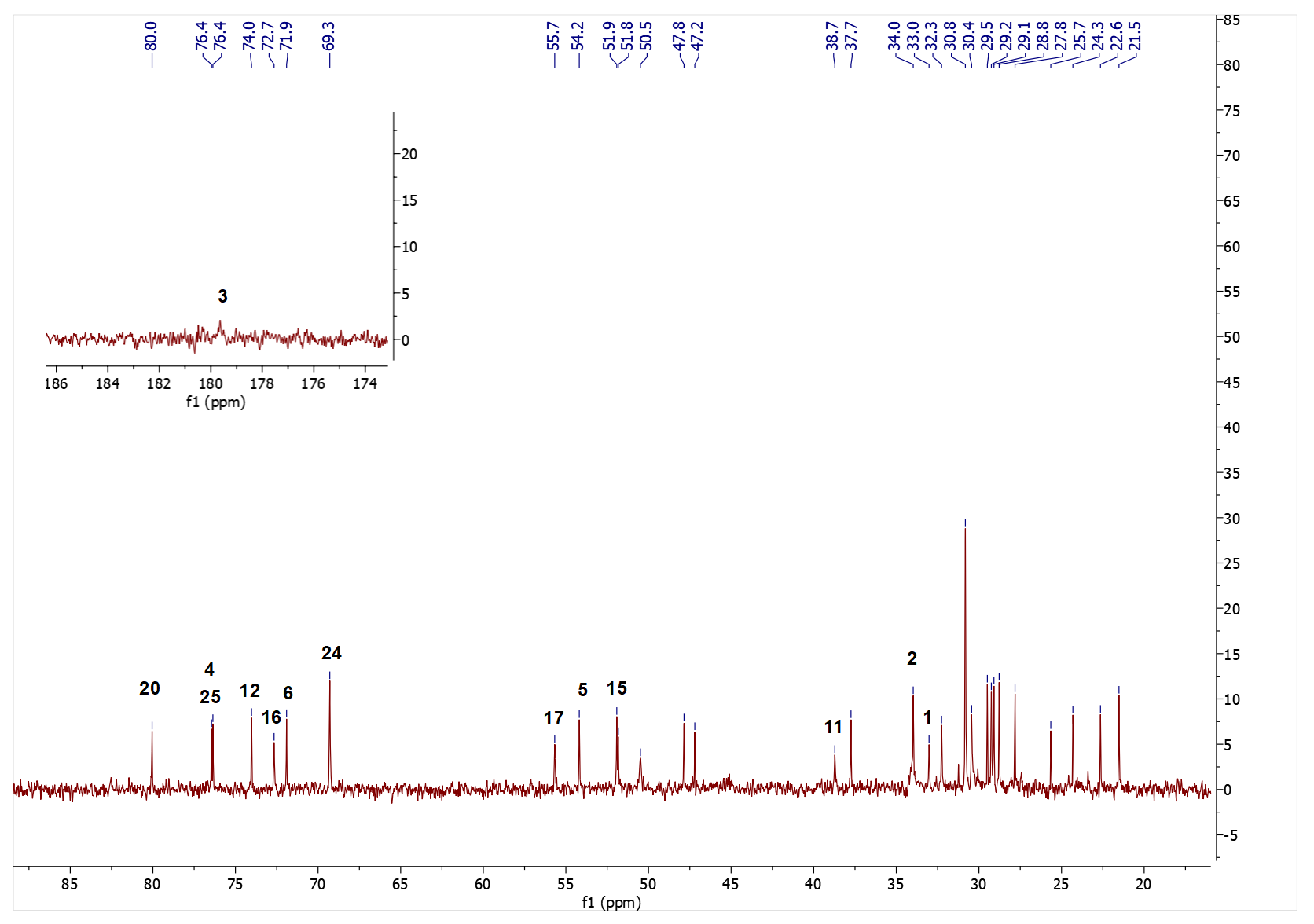


Fig. S 39. ^13^C-NMR spectrum of compound **9**.


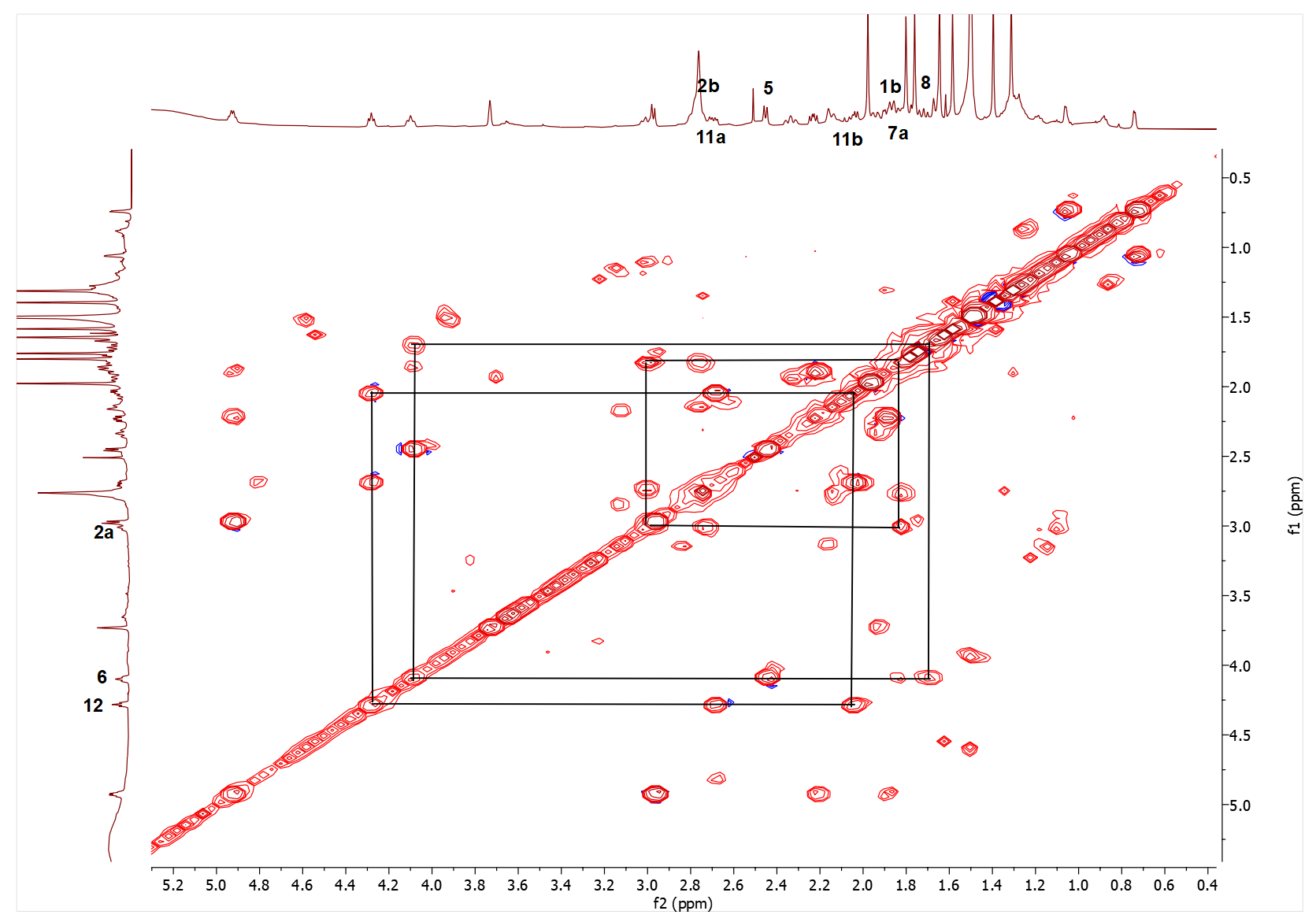


Fig. S 40. ^1^H-^1^H COSY spectrum of compound **9**.


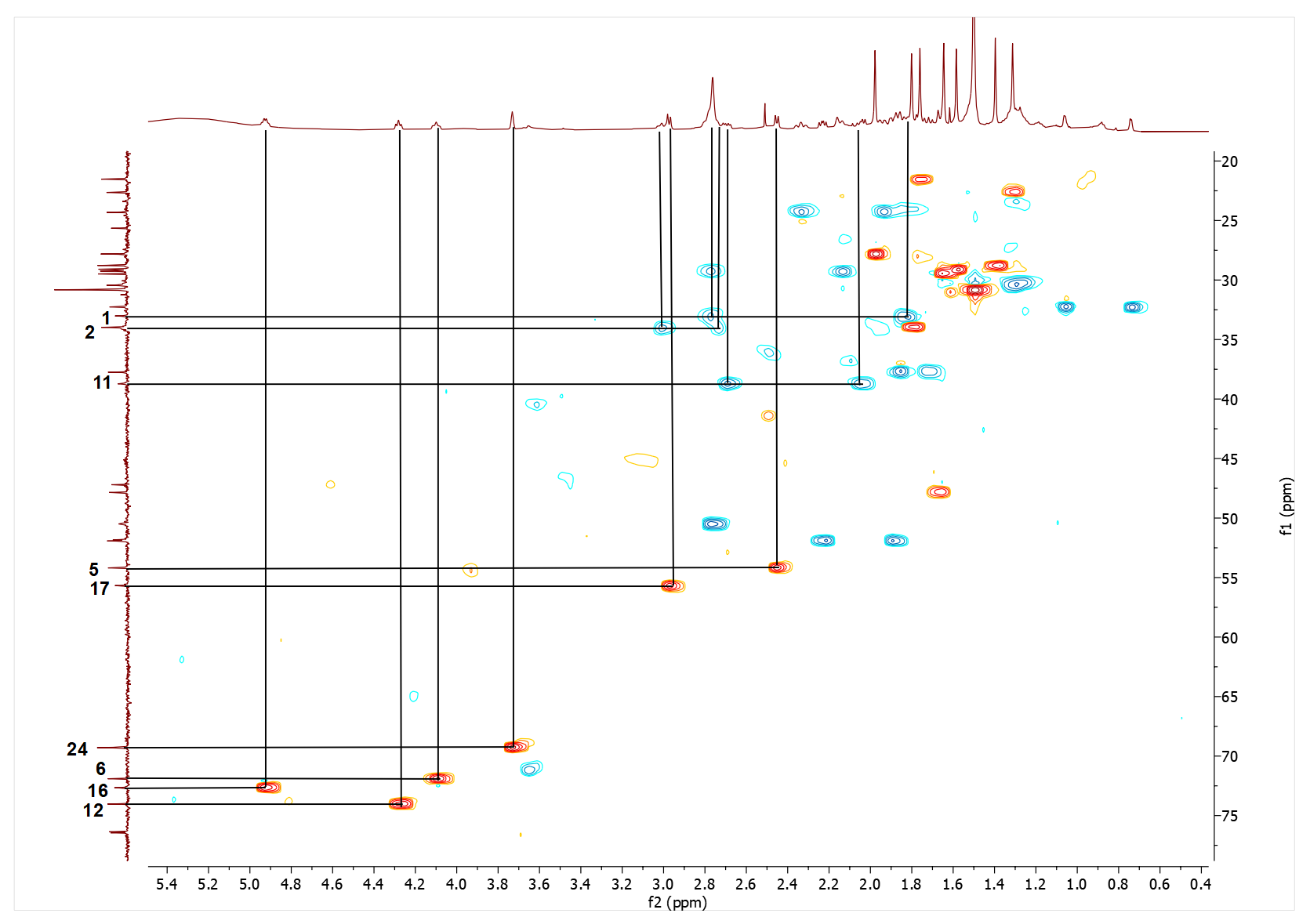


Fig. S 41. HSQC spectrum of compound **9**.


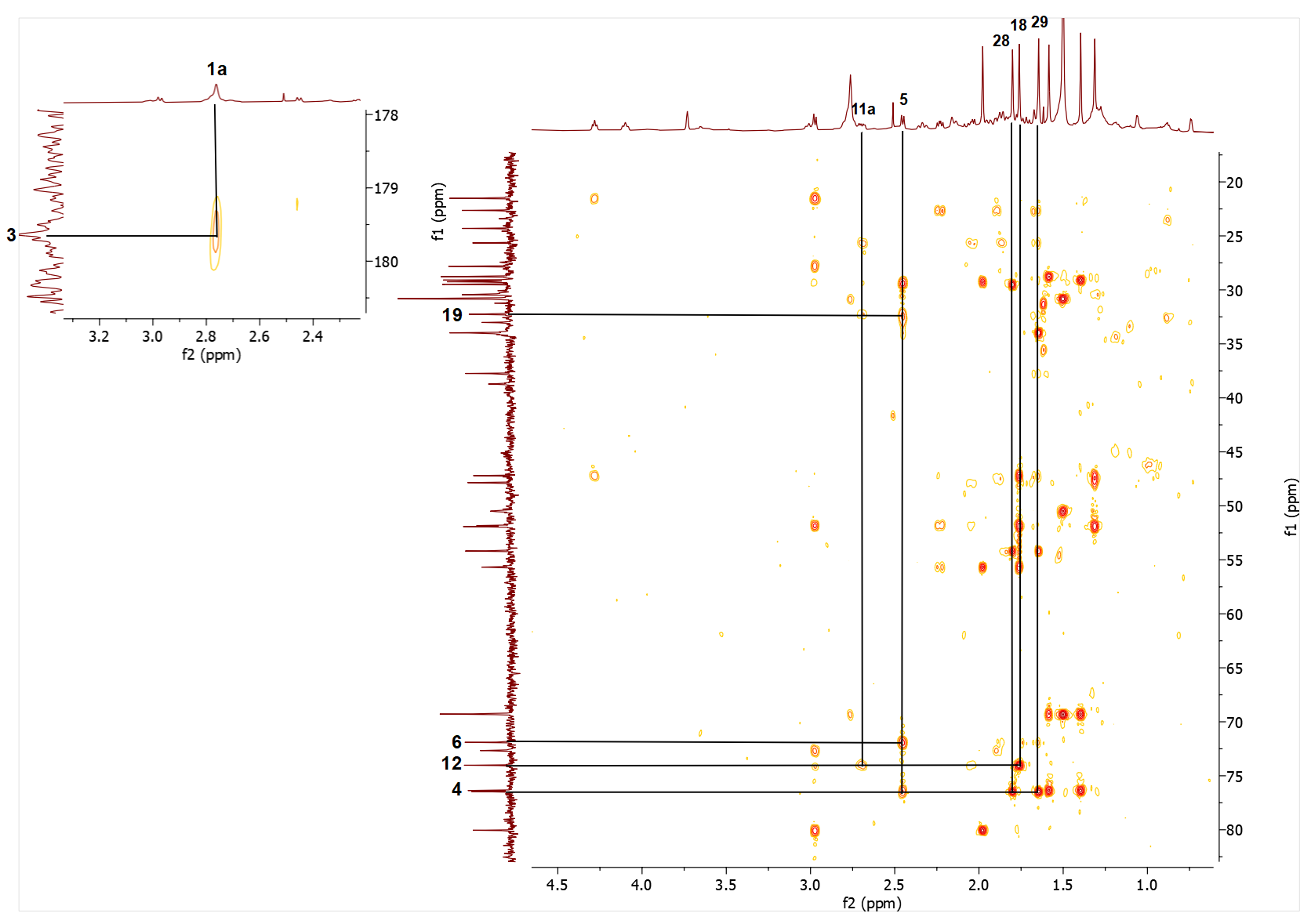

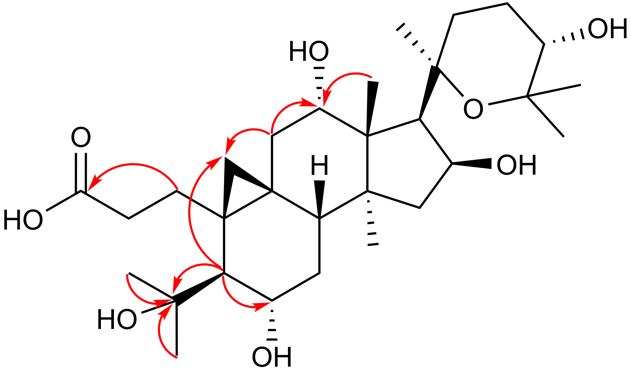


Fig. S 42. HMBC spectrum of compound **9**.

Fig. S 43. Chemical structure of compound **10**.


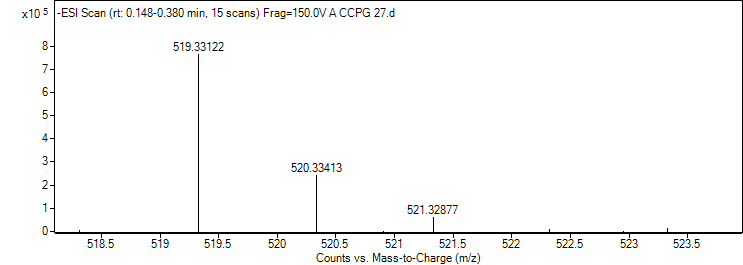


**[M-H]^-^**

Fig. S 44. HR-ESI-MS spectrum of compound **10**.


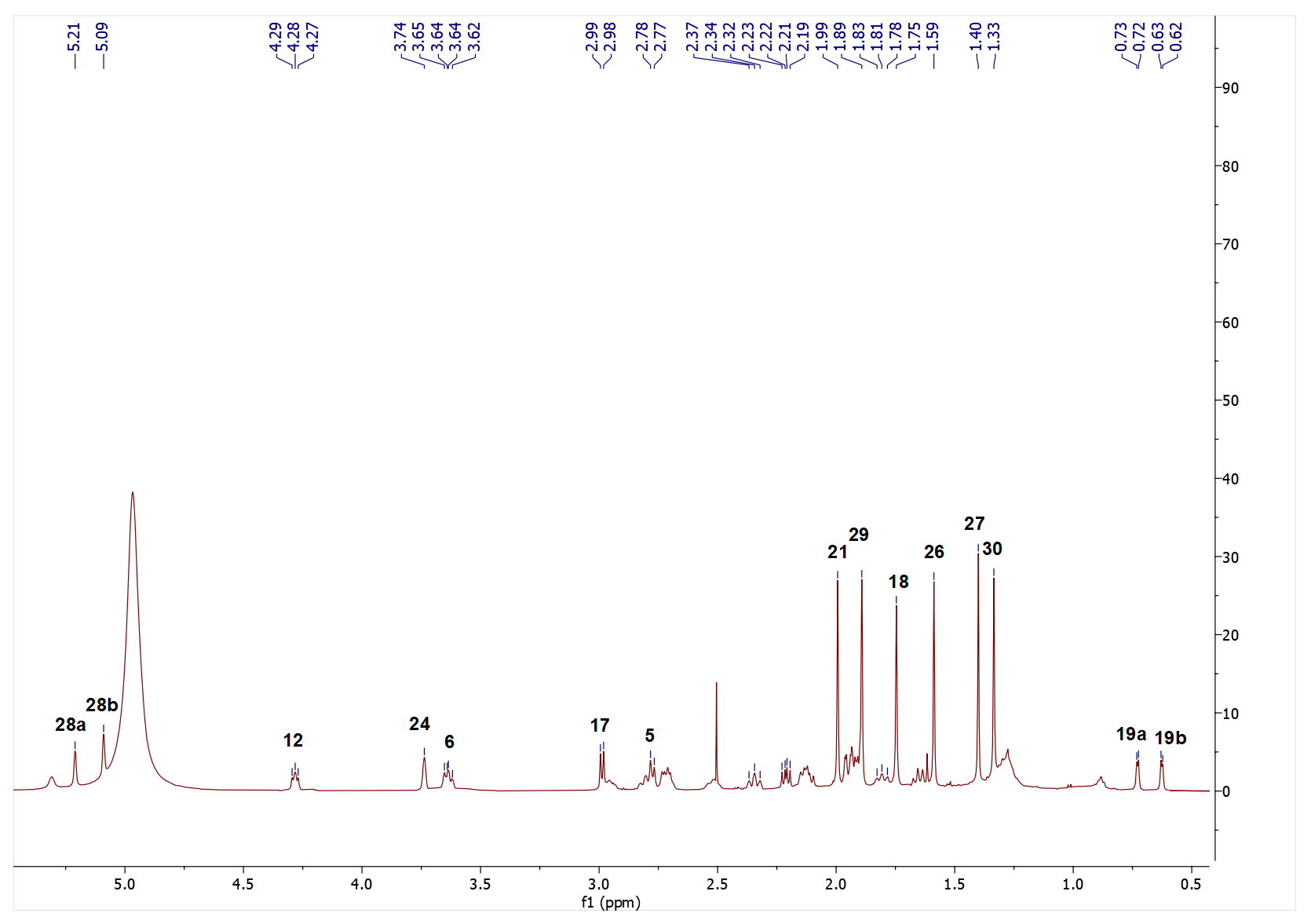


Fig. S 45. ^1^H-NMR spectrum of compound **10**.


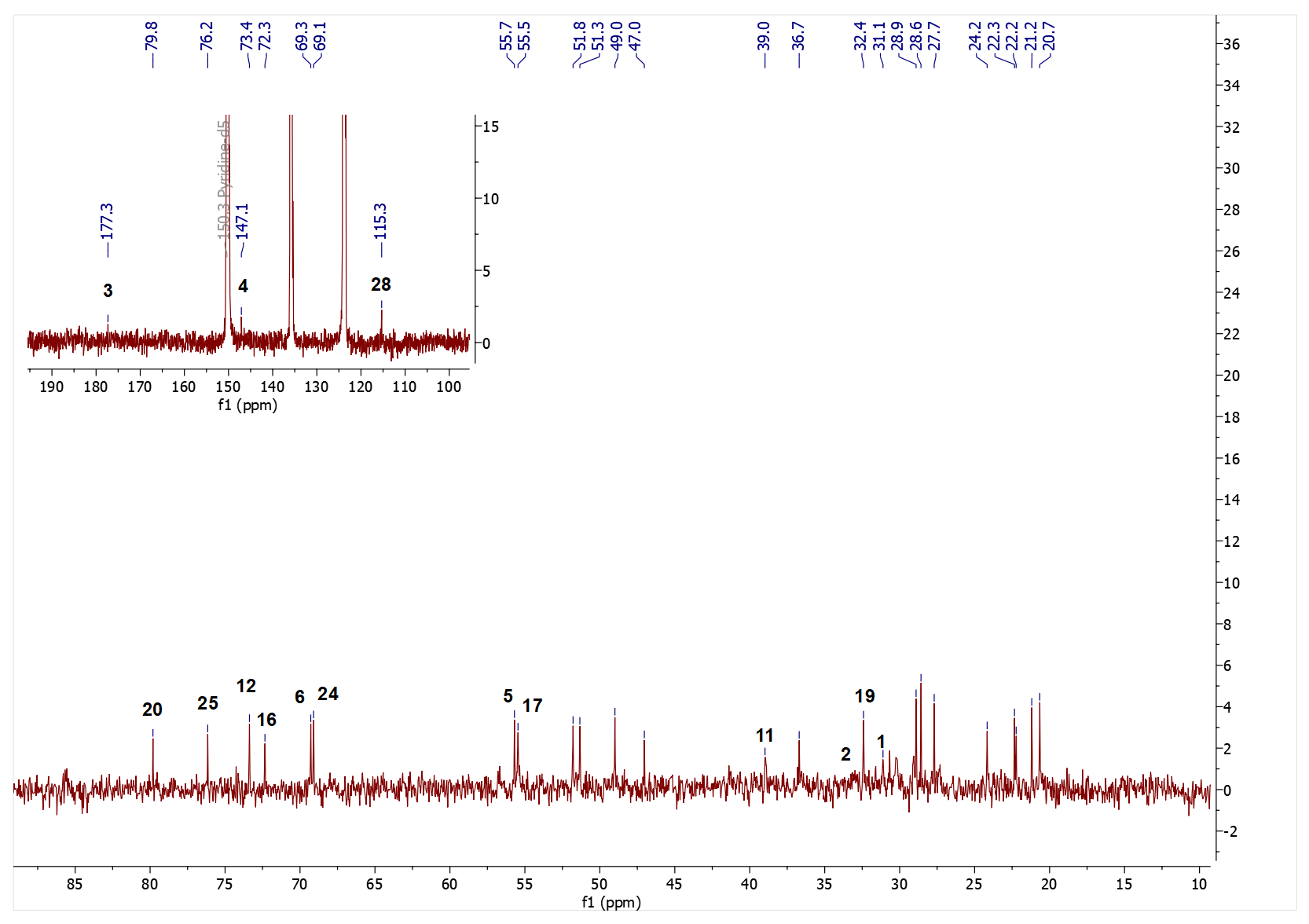


Fig. S 46. ^13^C-NMR spectrum of compound **10**.


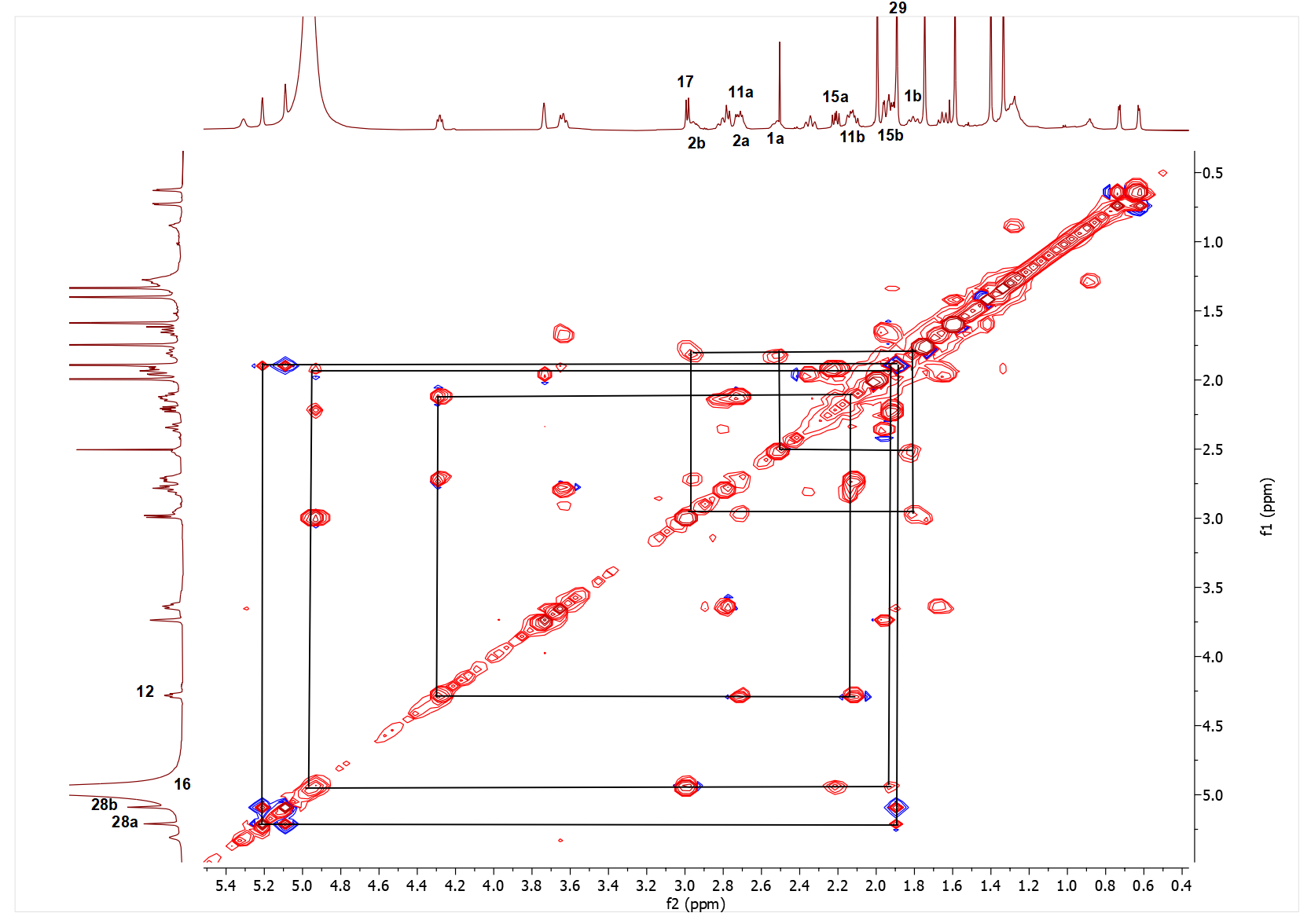


Fig. S 47. ^1^H-^1^H COSY spectrum of compound **10**.


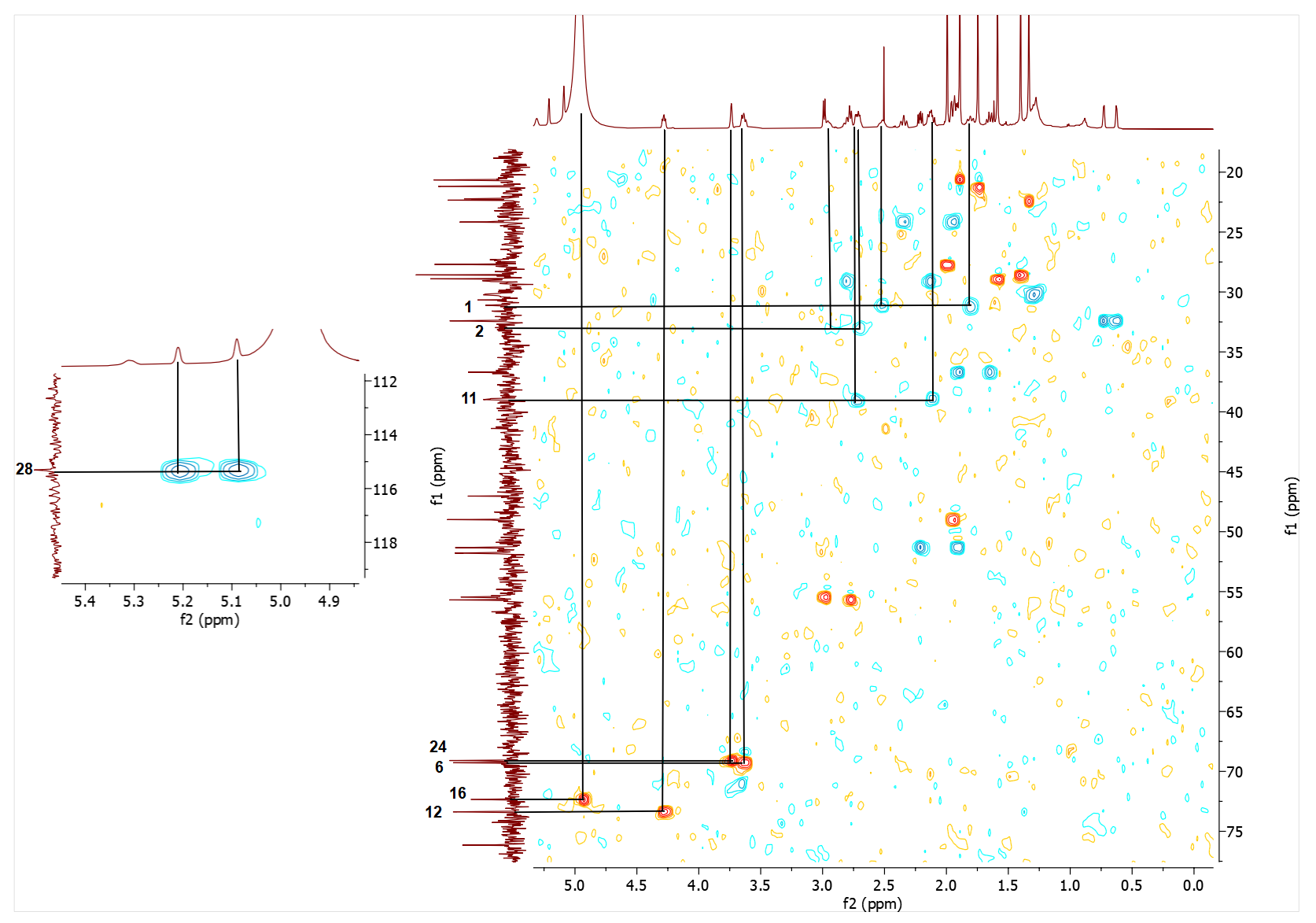


Fig. S 48. HSQC spectrum of compound **10**.


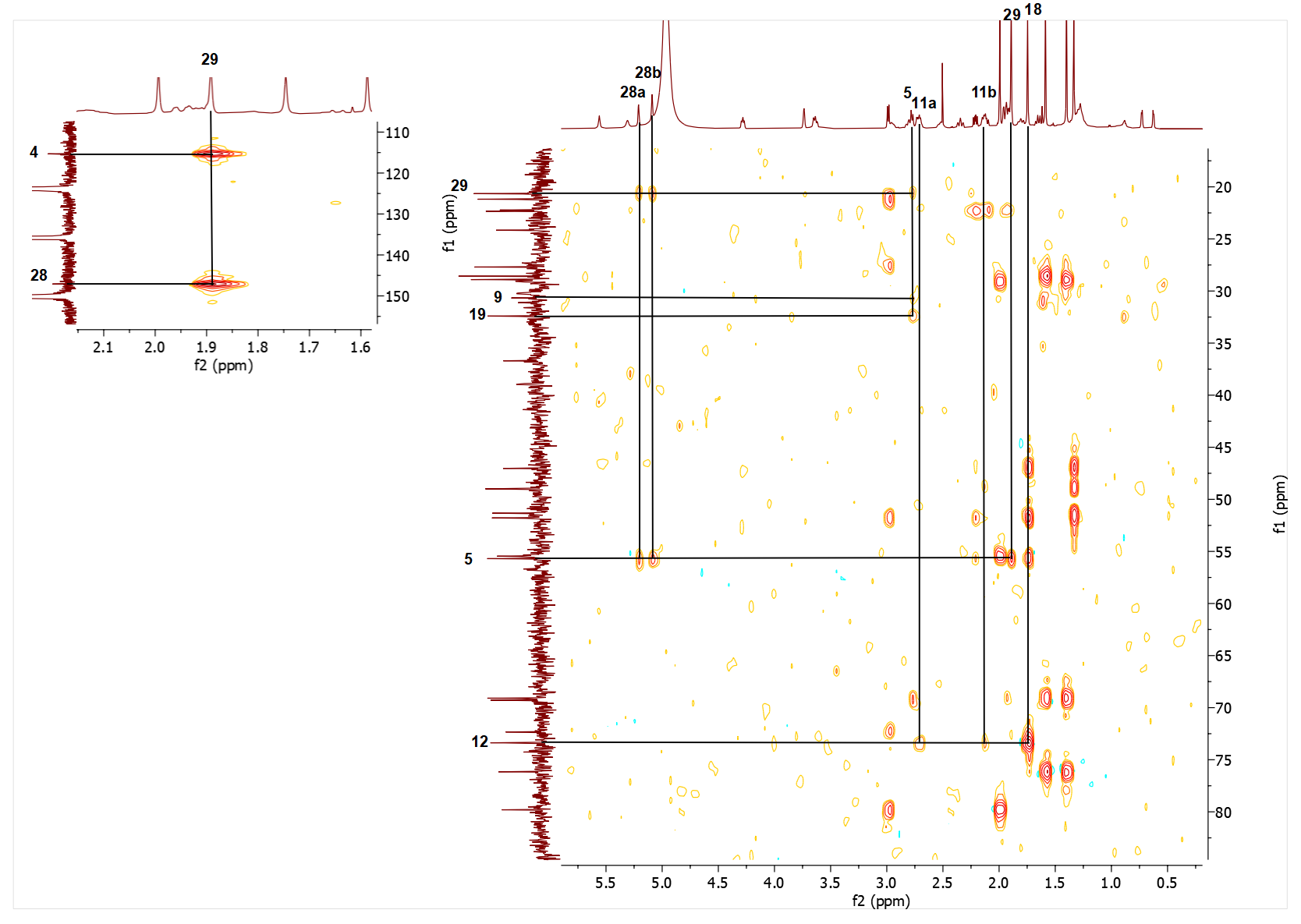

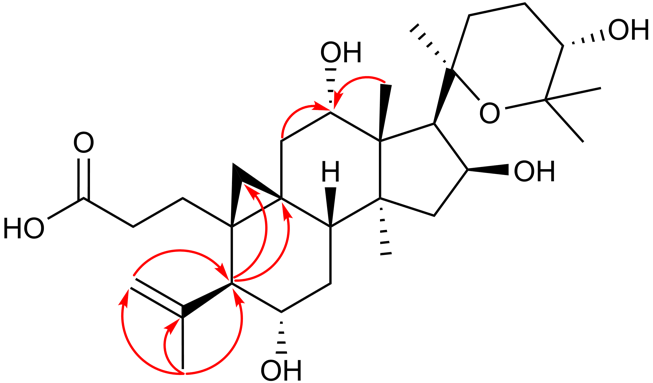


Fig. S 49. HMBC spectrum of compound **10**.
